# The ALDH2 gene rs671 polymorphism is associated with cardiometabolic risk factors in East Asian population: an updated meta-analysis

**DOI:** 10.3389/fendo.2024.1333595

**Published:** 2024-03-19

**Authors:** Ruikang Liu, Miaomiao Peng, Jiaoyue Zhang, Kangli Qiu, Tianshu Zeng, Lulu Chen

**Affiliations:** Department of Endocrinology, Union Hospital, Tongji Medical College, Huazhong University of Science and Technology, Wuhan, China

**Keywords:** ALDH2, meta-analysis, cardiometabolic risk factor, T2DM, hypertension

## Abstract

**Introduction:**

Acetaldehyde dehydrogenase 2 (ALDH2) had reported as a prominent role in the development of cardiometabolic diseases among Asians. Our study aims to investigate the relationship between ALDH2 polymorphism and cardiometabolic risk factors in East Asian population.

**Method:**

We searched databases of PubMed, Web of Science, and Embase updated to Oct 30^th^, 2023. We extracted data of BMI, Hypertension, SBP, DBP, T2DM, FBG, PPG, HbA1c, TG, TC, LDL-C and HDL-C.

**Result:**

In total, 46 studies were finally included in our meta-analysis, containing, 54068 GG and, 36820 GA/AA participants. All outcomes related to blood pressure revealed significant results (hypertension OR=0.83 [0.80, 0.86]; SBP MD=-1.48 [-1.82, -1.14]; DBP MD=-1.09 [-1.58, -0.61]). FBG showed a significant difference (MD=-0.10 [-0.13, -0.07]), and the lipid resulted significantly in some outcomes (TG MD=-0.07 [-0.09, -0.04]; LDL-C MD=-0.04 [-0.05, -0.02]). As for subgroups analysis, we found that in populations without severe cardiac-cerebral vascular diseases (CCVDs), GG demonstrated a significantly higher incidence of T2DM (T2DM OR=0.88 [0.79, 0.97]), while the trend was totally opposite in population with severe CCVDs (T2DM OR=1.29 [1.00, 1.66]) with significant subgroup differences.

**Conclusion:**

Our updated meta-analysis demonstrated that ALDH2 rs671 GG populations had significantly higher levels of BMI, blood pressure, FBG, TG, LDL-C and higher risk of hypertension than GA/AA populations. Besides, to the best of our knowledge, we first report GG had a higher risk of T2DM in population without severe CCVDs, and GA/AA had a higher risk of T2DM in population with severe CCVDs.

**Systematic Review Registration**

https://www.crd.york.ac.uk/PROSPERO, identifier CRD42023389242.

## Introduction

Acetaldehyde dehydrogenase 2 (ALDH2) in humans is a 517-amino acid polypeptide encoded by a nuclear gene located at chromosome 12q24 (1). Among the 19 kinds of human ALDH isozymes, ALDH2 is the most efficient Isozyme for metabolizing ethanol-derived acetaldehyde (2). Although ALDH2 is well-known for its crucial role in ethanol metabolism, ALDH2 also has other diverse pathophysiological effects: ALDH2 could also metabolize many other short-chain fatty aldehydes and some aromatic and polycyclic aldehydes, which provides essential protective enzyme functions against these toxic substances and potential mechanism of participating in the development of cardiovascular disease (CVD) (3, 4). Besides, recent research has revealed non-enzymatic functions of ALDH2, participating in lipid metabolism in hepatocytes, regulating foam cell formation in macrophages, and modulating cellular senescence in endothelial cells and vascular smooth muscle cells (5). ALDH2 A carriers have low ALDH2 enzyme activity, characterized by nausea, facial flushing, headache, palpitations and dizziness after drinking. This alcohol-induced ALDH2 A individual flushing syndrome is caused by a single G to A nucleotide change (also known as SNP rs671 G>A), substituting the glutamate to lysine at position 487 (see **Supplementary Figure S1**) (6). The ALDH2 A mutation has a dominant effect on the ALDH2 G allele, so the enzyme activity of homozygous ALDH2 AA is expected to be 1-4% enzyme activity of the wild-type, and enzyme activity of heterozygous ALDH2 GA is significantly lower than that of the wild-type by 50% (3).

CVD is a non-communicable disease responsible for a large number of deaths worldwide, and genetic factors have been proven to be one of the main risk factors (7, 8). Besides, Asians are more susceptible to metabolic disorders than other racial groups, regardless of obesity (9). However, only a limited number of specific genetic loci variations have been found between individuals of European and East Asian ancestry. Recently, several previously unreported variants among East Asian individuals had been reported related to type 2 diabetes (T2DM) in a comprehensive meta-analysis, which included 433,540 East Asian subjects from genome-wide association studies (GWAS) (10). Of these variants, ALDH2 rs671, which was estimated to be present in about 30–50% of the East Asian population, compared to less than 5% of the European population, was found to be most prominently associated with T2DM (11, 12).

Cardiometabolic risk factors encompass lifestyle habits and health conditions, such as obesity, dyslipidemia, hypertension, diabetes, and hyperuricemia (13). The interaction among these various cardiometabolic risk factors ultimately leads to the progression of cardiovascular diseases. In China, more than 40% of deaths are attributable to CVDs, and the number of CVD deaths has almost doubled in the past decades (14). The increasing prevalence of cardiometabolic risk factors underlies the rise of CVDs, and thus curbing the rising cardiometabolic pandemic is imperative (13). ALDH2 gene polymorphism had been widely studied in many human diseases (5, 9, 15, 16), but only a few research discussed the relationship between ALDH2 and cardiometabolic risk factors. One meta-analysis (Li et al., 2017) discussed the correlation between ALDH2 and T2DM with data from only six studies (17). Although it resulted in a significant conclusion, the sensitivity analysis and Egger’s test were positive, and the results between subgroups demonstrated an opposite trend of odds ratio (OR) value. In addition, the conclusions of several recently published articles were inconsistent with the meta-analysis (18, 19). Besides, the most recent meta-analysis discussing ALDH2 and hypertension (Zheng et. al., 2020) did not conduct any subgroup analysis (20). Meanwhile, ALDH2 was also reported in previous GWAS to be associated with hypertension, triglyceride (TG) and body mass index (BMI) (21). This revealed the prominent role of ALDH2 in the development of CVDs among Asians. Therefore, the meta-analysis to investigate the relationship between ALDH2 polymorphism and cardiometabolic risk factors in East Asian population needed to be updated.

## Methods

### Search strategy

Our protocol was prospectively registered in the International prospective register of systematic reviews (PROSPERO) registry (CRD42023389242). We followed the guidelines of Preferred Reporting Items for Systematic Reviews and Meta-Analyses (PRISMA) (**Supplementary Table S1**). Two independent reviewers (RL and PM) searched PubMed, Web of Science, and Embase (all updated to Oct 30^th^, 2023) and screened the titles and abstracts for potential included studies. The search strategy is available as **Supplementary Material** (**Supplementary Table S2**). We only full-text reviewed articles which met the inclusion criteria, and the reference lists of eligible articles were searched for additional citations.

### Selection criteria

There was no restriction on age, sex, and language. The inclusion criteria were (1): studies included subgroup data of ALDH2 gene rs671 polymorphism (GG, GA, AA type). (2) studies included at least one of the cardiometabolic risk factors: BMI, Hypertension, systolic blood pressure (SBP), diastolic blood pressure (DBP), T2DM, fasting blood glucose (FBG), postprandial blood glucose (PPG), hemoglobin A1c (HbA1c), TG, total cholesterol (TC), low-density lipoprotein cholesterol (LDL-C), high-density lipoprotein cholesterol (HDL-C). (3) studies only included the East Asian population. We excluded studies discussing other single nucleotide polymorphisms of ALDH2.

### Data extraction

Two researchers (RL and KQ) independently extracted general characteristics (number of participants, age, sex) and outcomes of cardiometabolic risk factors from eligible articles. Decisions were made by consulting another reviewer LC, when RL and KQ met disagreements and failed to reach a consensus. We emailed the corresponding authors for additional information when the data was incomplete.

### Quality assessment

Newcastle Ottawa Scale (NOS) was used for quality assessment of cohort and case-control studies (22), and the Agency for Healthcare Research and Quality (AHRQ) was used for cross-sectional studies (23). Two researchers (JZ and TZ) independently assessed the quality of included studies. The decision will be reached by consulting a third reviewer LC, when met disagreements and failed to reach a consensus. The precise Hardy Weinberg Equilibrium (HWE) was used to test the distribution of genotypes and evaluated by chi-square tests. High quality is considered NOS ≥6 points or AHRQ ≥8 points, and P_HWE_>0.05.

### Subgroup analysis

Several types of subgroup analysis were conducted in our study: (1) quality of study; (2) publication type (cross-sectional, cohort, case-control); (3) nationality (Chinese, Korean, Japanese); (4) population (with or without severe cardiac-cerebral vascular diseases (CCVDs), which including myocardial infarct, coronary artery disease, ischemic stroke and hemorrhagic stroke).

### Statistical analysis

Revman 5.3 and Stata 16.0 software were used to analyze Meta-analysis data. All forest plots and funnel plots were produced by Revman 5.3 software. Continuous data would be calculated by mean difference (MD) with 95% confidence intervals (CI). Dichotomous data would be calculated by OR with 95% CIs. Heterogeneity in the result of the meta-analysis was assessed by Cochrane Q and I^2^ statistics. I^2^ ≤ 50% demonstrated a low heterogeneity, and the fixed-effect model would be used for analysis; I^2^>50% demonstrated a high heterogeneity, and the random-effect model would be used for analysis. All statistical tests were two-tailed, and P ≤ 0.05 was regarded as a statistically significant difference. Publication bias was assessed by funnel plots and Egger’s tests. Sensitivity analysis was performed in the meta-analysis by excluding each study once at a time to check whether the effectiveness of the outcome was determined by individual studies.

## Result

### Characteristics of meta-analysis

**Figure 1:**
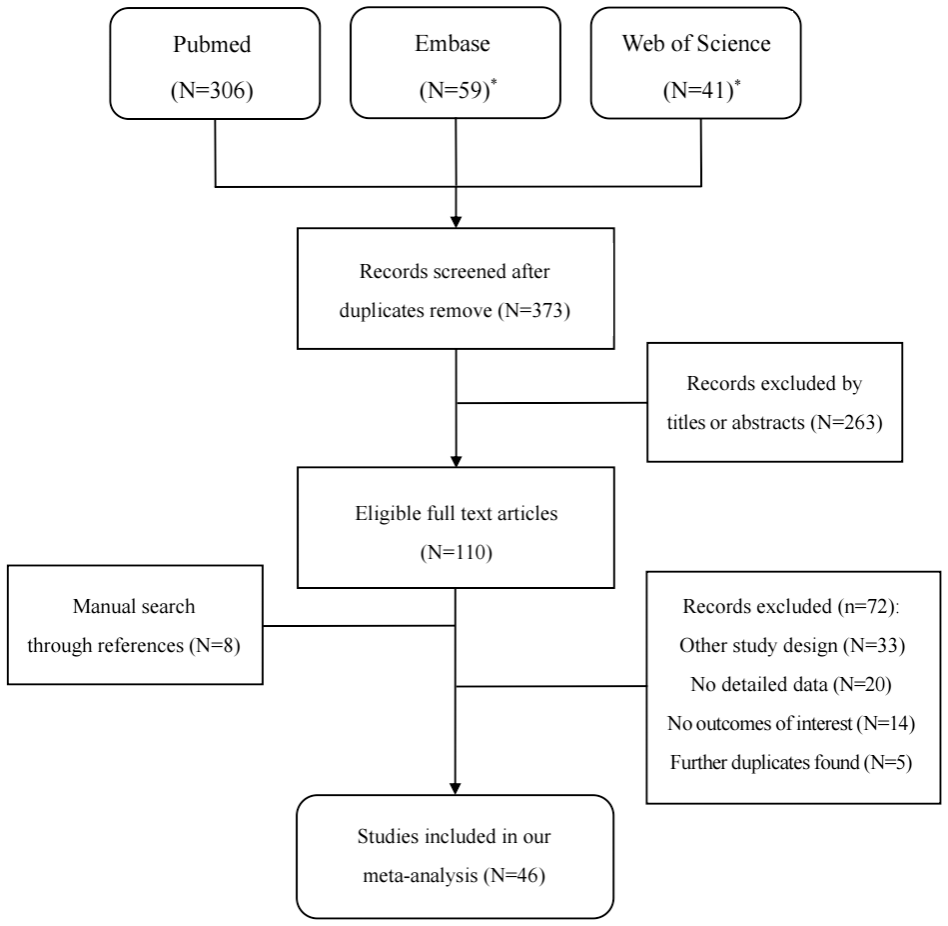
Flowchart of selection of studies for inclusion in our meta-analysis. (*Outcomes from Embase and Web of science were not included studies from Medline).

The detailed steps of the literature search are shown in **Figure 1**: 373 studies were reviewed from three databases, and eight studies were reviewed through the references of eligible full-text articles; 263 studies were excluded after screening titles and abstracts, and the remaining 118 studies were reviewed in full text. After excluding 72 studies according to the selection criteria, 46 studies with, 54068 GG participants and, 36820 GA/AA participants were included in our meta-analysis (19, 20, 24–67).

The general characteristics of included studies in our meta-analysis are shown in **Table 1**. 23 studies were Chinese, 22 were Japanese, and one was Korean. As for publication type, 17 were cross-sectional studies, 13 were cohort studies, and 16 were case-control studies. Among all 46 studies, 36 studies (78.3%) included populations without severe CCVDs, and 35 studies (76.1%) were assessed as high quality according to HWE and NOS or AHRQ. Detailed procedures of NOS and AHRQ are shown in **Supplementary Table S3**.

**Table 1:** General characteristics of included studies in our meta-analysis.

Year Author^a^	Nationality	Population^c^	Article type	Gender (M/F)	GG	GA	AA	F(G)	F(A)	HWE	NOS/AHRQ
2023 Hisamatsu	Japanese	Normal	Cross-sectional	All Male	369	255	58	0.728	0.272	0.345	11
2023 Hyashida	Japanese	Normal	Cross-sectional	4572/6338	6131	4779	/	N/A			9
2023 Lan	Chinese	EH	Case-control	3057/1474	2123	2001	407	0.689	0.311	0.111	9
2023 Okura	Japanese	Normal	Cross-sectional	41/30	38	30	3	0.746	0.254	0.618	8
2023 Zhang	Chinese	HS^d^	Case-control	214/115	184	123	22	0.746	0.254	0.973	7
2022 Wu^b^	Chinese	EH	Case-control	2040/1017	1442	1358	257	0.694	0.306	0.042	9
2022 Li	Chinese	Normal	Prospective cohort	3468/2700	3105	3063		N/A			8
2021 He	Chinese	Normal	Prospective cohort	1110/1390	1571	801	128	0.789	0.211	0.134	8
2021 Ishida	Japanese	MI^d^	Retrospective cohort	158/60	98	106	14	0.693	0.307	0.111	6
2021 Kogiso	Japanese	NAFLD	Case-control	161/153	160	154		N/A			6
2021 Takeno	Japanese	Normal	Prospective cohort	All Male	53	35	6	0.750	0.250	0.998	6
2021 Zhu	Chinese	MI^d^	Case-control	81/51	273	263	64	0.674	0.326	0.998	7
2020 Hou	Chinese	MI^d^	Retrospective cohort	637/326	502	370	91	0.713	0.287	0.172	7
2020 Kim	Korean	Normal	Retrospective cohort	3319/5207	5880	2424	222	0.832	0.168	0.332	7
2020 Wang^b^	Chinese	Normal	Cross-sectional	8431/4670	8891	3857	353	0.826	0.174	0.027	9
2020 Zhu	Chinese	Normal	Cross-sectional	250/299	322	227		N/A			9
2019 Han^b^	Chinese	Normal	Cross-sectional	All Male	376	80	11	0.891	0.109	0.036	7
2019 Xia^b^	Chinese	Normal	Cross-sectional	389/224	379	213	21	0.792	0.208	0.400	7
2017 Li	Chinese	EH	Case-control	1420/1798	1791	1090	157	0.768	0.232	0.820	8
2017 Ma	Chinese	Normal	Cross-sectional	2805/1213	2759	1152	107	0.830	0.170	0.596	10
2017 Zhu	Chinese	MetS	Case-control	N/A	2003	2292		N/A			9
2016 Mizuno^b^	Japanese	MI^d^	Retrospective cohort	156/46	99	86	17	0.703	0.297	0.962	5
2016 Oniki	Japanese	Normal	Retrospective cohort	196/145	202	118	21	0.765	0.235	0.797	8
2016 Sung^b^	Chinese	IS^d^	Case-control	All Male	308	223	67	0.702	0.298	0.028	7
2016 Yin	Japanese	Normal	Cross-sectional	907/911	1046	648	124	0.754	0.246	0.229	10
2015 Idewaki	Japanese	Normal	Cross-sectional	2483/1917	2402	1655	343	0.734	0.266	0.051	8
2015 Qu	Chinese	IS^d^	Retrospective cohort	111/48	100	52	7	0.792	0.208	0.997	7
2015 Taylor	Chinese	T2DM	Case-control	1352/2400	2202	1334	216	0.765	0.235	0.759	8
2015 Yokoyma	Japanese	Normal	Cross-sectional	All Male	1528	278	/	N/A			9
2013 Lv	Chinese	EH	Case-control	300/165	282	169	14	0.788	0.212	0.162	7
2013 Morita	Japanese	DR	Case-control	156/78	143	78	13	0.778	0.222	0.861	7
2013 Wang	Chinese	EH	Case-control	1266/853	1228	769	122	0.761	0.239	0.994	8
2013 Yokoyama	Japanese	Normal	Cross-sectional	All Male	1605	297		N/A			8
2012 Feng	Chinese	EH	Case-control	91/20	53	24	3	0.812	0.188	0.991	7
2011 Hasi	Chinese	EH	Case-control	81/80	138	23	0	0.929	0.071	0.621	7
2010 Xu	Chinese	CAD^d^	Case-control	157/152	516	306	29	0.786	0.214	0.129	7
2008 Dakeishi	Japanese	Normal	Cross-sectional	492/183	519	143	13	0.875	0.125	0.696	9
2007 Hui	Japanese	EH	Case-control	352/180	302	195	35	0.751	0.249	0.898	7
2007 Nagawasa^b^	Japanese	Normal	Cross-sectional	159/217	237	133	6	0.807	0.193	0.030	10
2007 Xu	Chinese	All CAD^d^	Cross-sectional	186/45	145	77	9	0.794	0.206	0.953	8
2004 Suzuki^b^	Japanese	Normal	Retrospective cohort	114/44	85	73		N/A			4
2003 Saito	Japanese	Normal	Cross-sectional	All Male	177	137	21	0.733	0.267	0.719	8
2002 Amamoto	Japanese	Normal	Cross-sectional	749/1286	1056	906	150	0.714	0.286	0.061	8
2002 Takagi^b^	Japanese	All MI^d^	Retrospective cohort	All Male	1035	925	202	0.693	0.307	0.975	5
2000 Murata^b^	Japanese	Normal	Retrospective cohort	All Male	90	63	10	0.745	0.255	0.973	4
1996 Suzuki^b^	Japanese	Normal	Retrospective cohort	127/85	120	80	12	0.755	0.245	0.962	5

N/A, not applicable; HWE, Hardy Weinberg Equilibrium; NOS, Newcastle Ottawa Scale; EH ,essential hypertension; MI, myocardial infarct; CAD, coronary disease; IS, ischemic stroke; HS, hemorrhagic stroke; MetS, metabolic syndrome; NAFLD, nonalcoholic fatty liver disease; DR, diabetic retinopathy; AHRQ, Agency for Healthcare Research and Quality; F(G), frequency of G gene. ^a^Search result updated to Oct 30^th^, 2023. ^b^These studies meet one of the criteria: (1) P(HWE)≤0.05; (2) assessed as low quality of NOS or AHRQ. We compare the forest plot with vs. without these studies, the significance of P values are the same, so they are included in our meta-analysis. ^c^Normal population means without severe CCVDs in their included participants. ^d^These populations are regarded as participants with severe CCVDs, and they are separated as a subgroup in the meta-analysis.

### Outcomes of meta-analysis

**Figure 2:**
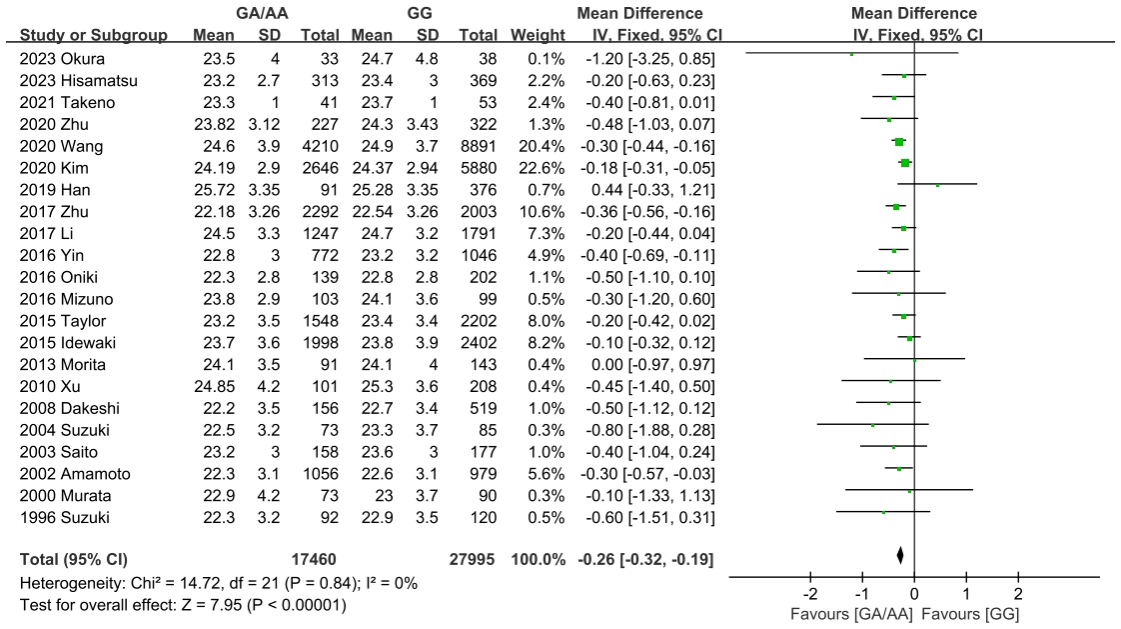
Forest plot of ALDH2 GA/AA vs. GG on BMI.

**Figure 3:**
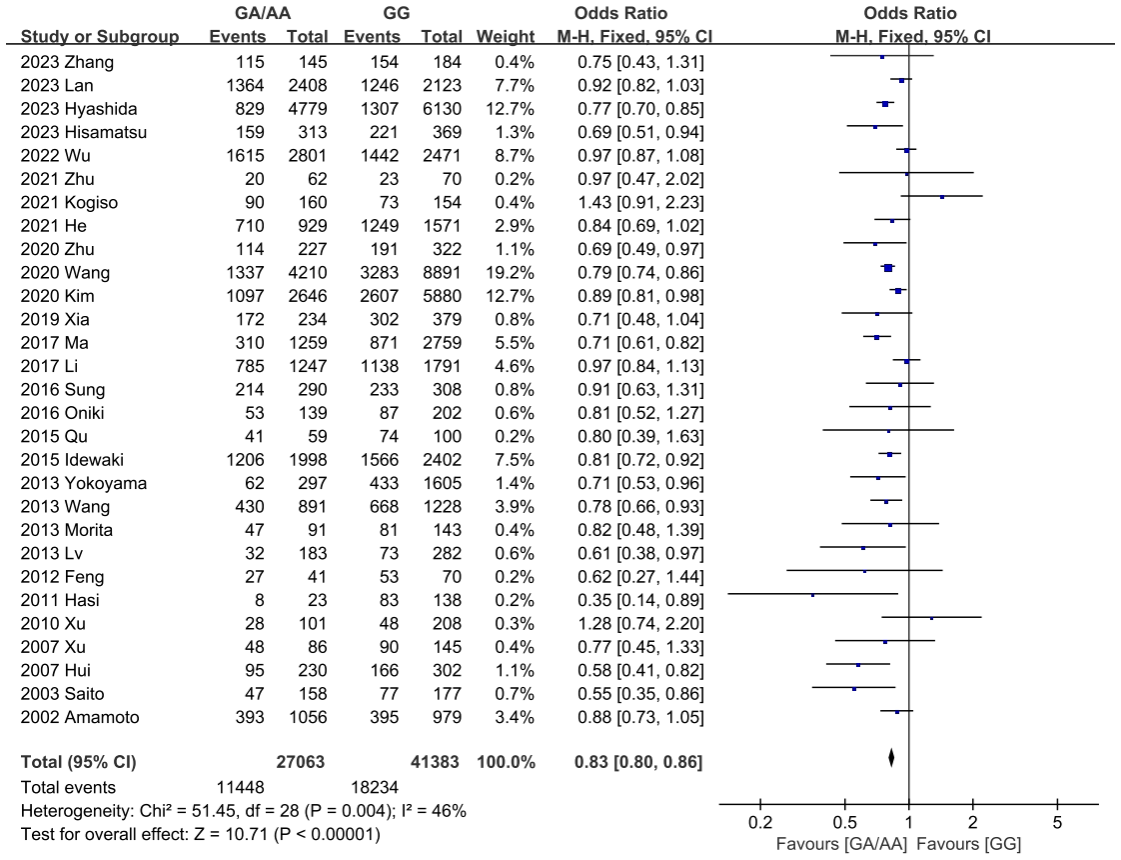
Forest plot of ALDH2 GA/AA vs. GG on incidence of hypertension.

**Figure 4:**
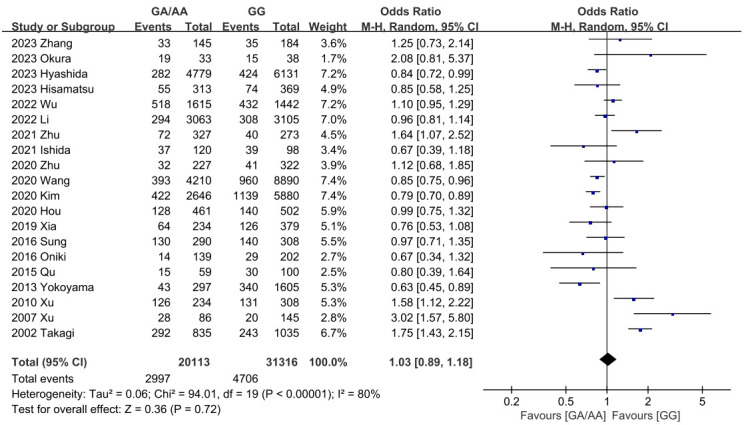
Forest plot of ALDH2 GA/AA vs. GG on incidence of T2DM.

Summary findings of outcomes are shown in **Table 2**. Firstly, the differences of all confounders in our study were insignificant. It was worth mentioning that ALDH2 had a significantly different effect on T2DM, so the P_gender_>0.05 could eliminate the bias caused by gender. As for outcomes, the GG population had demonstrated a significantly higher BMI level than the GA/AA population (MD=-0.26 [-0.32, -0.19], P<0.001, detailed forest plot see **Figure 2**). Besides, all outcomes related to blood pressure revealed significant results (incidence of hypertension OR=0.83 [0.80, 0.86], P<0.001; SBP MD=-1.48 [-1.82, -1.14], P<0.001; DBP MD=-1.09 [-1.58, -0.61], P<0.001; detailed forest plot of hypertension see **Figure 3**). However, only FBG showed a significant difference (MD=-0.10 [-0.13, -0.07], P<0.001), while the incidence of T2DM resulted in an insignificant difference (detailed forest plot see **Figure 4**). Moreover, the lipid resulted significantly in some of the outcomes (TG MD=-0.07 [-0.09, -0.04], P<0.001; LDL-C MD=-0.04 [-0.05, -0.02]). In all, ALDH2 is significantly associated with the most cardiometabolic risk factors in the total population. All detailed forest plots are displayed in **Supplemental Files** (see **Supplementary Figures S2**-**S12**). Funnel plots of BMI, hypertension and T2DM are also showed in **Supplementary Figures S13**-**15**. Egger’s test was only significant in the outcome of HbA1c. All outcomes of the sensitivity analysis were also insignificant.

**Table 2:** Summary findings of primary outcomes.

Characteristic	No. of study	Participants	Statistical method	95% CI	I2 (%)^a^	P value	Egger's test
Confounders							
Age	23	42614	MD	0.29 [-0.09, 0.67]	66	0.13	0.139
Gender	21	57139	OR	1.04 [0.96, 1.14]	72	0.32	0.086
Smoker	22	39990	OR	1.01 [0.92, 1.10]	55	0.86	0.925
Outcomes							
BMI	22	45455	MD	-0.26 [-0.32, -0.19]	0	<0.001*	0.103
Hypertention	29	68446	OR	0.83 [0.80, 0.86]	46	<0.001*	0.211
SBP	17	46748	MD	-1.48 [-1.82, -1.14]	48	<0.001*	0.782
DBP	17	46748	MD	-1.09 [-1.58, -0.61]	71	<0.001*	0.331
T2DM	20	51429	OR	1.03 [0.89, 1.18]	80	0.72	0.273
FBG	19	35154	MD	-0.10 [-0.13, -0.07]	46	<0.001*	0.666
HbA1c	10	6635	MD	0.03 [-0.01, 0.07]	0	0.19	0.018*
TC	16	36984	MD	-0.02 [-0.04, 0.00]	37	0.08	0.869
TG	20	43419	MD	-0.07 [-0.09, -0.04]	54	<0.001*	0.509
LDL-C	17	37961	MD	-0.04 [-0.05, -0.02]	0	<0.001*	0.834
HDL-C	19	42737	MD	-0.02 [-0.04, 0.01]	91	0.25	0.826

BMI, body mass index; SBP, systolic pressure; DBP, diastolic pressure;T2DM, type 2 diabetes mellitus; FBG, fast blood glucose; HbA1c, glycosylated haemoglobin A1c; TC, total cholesterol; TG, triglyceride; LDL-C, low-density lipoprotein cholesterol; HDL-C, high-density lipoprotein cholesterol; MD, mean difference; OR, odds ratio; CI, confidence interval. ^a^I^2^≤50% used fixed-effect model for analysis; I^2^>50% used random-effect model for analysis. *P≤0.05.

### Subgroup analysis

There were four types of subgroup analyses in our study. **Supplementary Table S4** shows that all subgroup differences between different qualities of included studies were insignificant, so the studies of low qualities were not excluded from our meta-analysis. **Supplementary Table S5** shows that all subgroup differences between different nationalities of included studies were also insignificant. Besides, **Supplementary Table S6** demonstrates that all subgroup differences between different article types were insignificant. However, we found that in populations without severe CCVDs, GG demonstrated a significantly higher incidence of T2DM and FBG level (GA/AA vs. GG: T2DM OR=0.88 [0.79, 0.97]; MD=-0.11 [-0.14, -0.08]), while the trend was totally opposite in population with severe CCVDs (GA/AA vs. GG: OR=1.29 [1.00, 1.66]; MD=0.25 [0.09, 0.41]) with significant subgroup differences (P_T2DM_=0.006, P_FBG_<0.001, detailed data see **Supplementary Table S7**). Moreover, other outcomes revealed insignificant subgroup differences. It is worth mentioning that the subgroup analysis between severe CCVDs and without severe CCVDs was the first time reported.

## Discussion

Our meta-analysis indicated that ALDH2 rs671 GG gene type was not only a risk factor for promoting the development of hypertension, but also in increasing BMI, blood pressure, FBG, TG, and LDL-C. In addition, the impact of ALDH2 on T2DM and FBG varies significantly among different populations. In the population without severe CCVDs, the incidence of T2DM and FBG levels in the GG population were significantly higher than the GA/AA population. In contrast, the conclusion was totally opposite in the population with severe CCVDs. Our results were more comprehensive and convincing based on the following aspects: Firstly, our meta-analysis included more eligible studies, providing sufficient statistical efficacy. Secondly, confounding factors were examined in our meta-analysis to avoid the potential intervention. Thirdly, we conducted four subgroup analyses on factors that may affect the reliability to reduce the heterogeneity between different studies. Fourthly, sensitivity analysis and Egger’s test showed that the results were stable and reliable, and no significant publication bias was found.

Currently, there is no meta-analysis discussing the relationship between ALDH2 and BMI, and our meta-analysis demonstrated that ALDH2 GG populations had a significantly higher BMI than GA/AA populations in the outcome of all subgroups. Some clinical articles have reported lower BMI due to healthier dietary habits caused by flushing syndrome in the GA/AA population (68, 69). Besides, it should be clarified that the correlation between ALDH2 and BMI varies among different genders. Gender differences also exist in other cardiometabolic outcomes (10, 70). Several studies have reported the activation effect of estradiol on ALDH2 (71, 72). Besides, we cannot ignore the differences in alcohol consumption between different genders due to social factors in East Asian culture, as well as the protective effect of estrogen itself on insulin resistance (73).

T2DM and hypertension are common microvascular and macrovascular diseases in East Asian populations (74). ALDH2 mutants have lower levels of BMI, providing a partial explanation for the correlation between ALDH2 polymorphism and T2DM and hypertension. In addition, other literature also discussed the mechanisms between ALDH2 and cardiometabolic risk factors. Previous studies on ALDH2 rs671 polymorphism and T2DM had provided controversial results (25, 35, 38, 40, 57, 63, 67). This may be due to the sample size of most ALDH2 studies being relatively small (28 included studies of our study had participants less than, 1000), and ALDH2 was interfered with other confounding factors. Our study included abundant studies and analyzed the confounders (age, gender, and smoker) to avoid the potential bias. Besides, it was worth mentioning that ALDH2 rs671 polymorphism was reported to modify the association between dietary behaviors and BMI independently of drinking habits. Therefore, lower BMI and alcohol intake of GA/AA individuals were supposed to be the mechanism of ALDH2 on T2DM in populations without severe CCVDs. However, people with severe CCVDs revealed a higher risk of T2DM and FPG levels in the GA/AA population. Some mechanisms may explain it (1): Tan et al. demonstrated that ALDH2 alleviated the ischemia and reperfusion injury in diabetic cardiomyopathy through inhibition of mitoPTP opening and activation of PI3K/AKT/mTOR pathway (75); (2) low ALDH2 activity exacerbated 4HNE-mediated coronary endothelial cell injury and thereby cardiac dysfunction and ischemia-reperfusion injury (76, 77). A meta-analysis by Xu et al. also concluded the correlation between the higher risk of ischemic stroke and ALDH2 rs671-variant, especially in AA populations. Therefore, for CAD populations, GA/AA individuals had a higher risk of progressing to severe CCVDs, which explained the opposite outcomes in the severe CCVDs subgroup. Moreover, Chang et al. demonstrated that male Aldh2 knock-in mice were prone to develop glucose intolerance, insulin resistance, and fatty liver under diet-induced obesity. Proteomic analyses of the brown adipose tissue from the male Aldh2 knock-in mice identified increased 4-hydroxynonenal-adducted proteins involved in mitochondrial fatty acid oxidation and electron transport chain, leading to markedly decreased fatty acid oxidation rate and mitochondrial respiration. Similar phenomena were also reproduced in other studies of mice experiments (78, 79). In summary, we inferred that in the normal population, ALDH2 GG has a higher risk of T2DM due to higher BMI and alcohol consumption, but severe CCVDs contributed to exacerbating central muscle cell damage in the GA/AA individuals with a higher FBG level.

As for the hypertension-related results, a statistically significant correlation between ALDH2 and hypertension was observed in all subgroups. Therefore, we could confirm that the GG population had a higher risk of hypertension and higher levels of SBP and DBP as well. Although GA/AA individuals had more acetaldehyde accumulation, Zhang et al. reported in their cohort study that ethanol, rather than acetaldehyde, played a key role in alcohol-induced hypertension (80). Therefore, GA/AA individuals were protected from hypertension due to their lower ethanol intake than GG individuals. In addition, though our conclusion was consistent with the recently published meta-analysis by Zheng et al., we had updated hypertension-related data by adding from twelve new studies than their study (20). Moreover, we analyzed confounders (age and smoker) and three new subgroup analyses, which they did not include, so we believed our article was updated compared to previous studies.

ALDH2 also revealed a significant correlation with TG and LDL-C. TG is the primary dietary lipid, so the level of TG is almost only influenced by dietary habits (81). The higher TG level of GG populations demonstrated unhealthier dietary habits than GA/AA populations. In recent years, many researchers have tried to explain the differences between ALDH2 gene types and lipids in other mechanisms. Gibb et al. found that the ALDH2 rs671 mutant could repress the transcription of a lysosomal H+ pump subunit in nucleus, which is crucial for lipid degradation and foam cell formation (82). Besides, Zhong et al. also discovered an unexpected interaction of ALDH2 with the LDL receptor, which may directly act on ATP6V0E2 (a critical substance for maintaining lysosomal function and degradation of oxidized LDL-C) and increase foam cell formation (83). However, the correlation between ALDH2 and TG or LDL-C was not strong enough that not all outcomes were significant in the subgroups. Further studies of ALDH2 including lipid-related data were needed.

In all, ALDH2 is primarily involved in the degradation of acetaldehyde which reduced ALDH2 activity could lead to “Asian flush syndrome,” affecting alcohol intake. Additionally, ALDH2 could also metabolize endogenous lipid aldehydes. Reduced ALDH2 activity results in lipid aldehyde accumulation, generating reactive oxygen species and activating various oxidative stress pathways. Over the past few decades, numerous studies have shown the dual effect between ALDH2 gene polymorphisms and cardiovascular diseases: ALDH2 mutation could increase the risk of coronary artery disease, peripheral artery disease and stroke; ALDH2 mutation could also decrease the risk of hypertension and aortic aneurysms or dissections (5). Besides, recent research has revealed non-enzymatic functions of ALDH2, participating in lipid metabolism in hepatocytes, regulating foam cell formation in macrophages, and modulating cellular senescence in endothelial cells and vascular smooth muscle cells. Moreover, our meta-analysis concludes that ALDH2 mutants had lower BMI levels. In summary, ALDH2 has diverse pathophysiological effects, and ALDH2 mutations can variably influence cardiovascular metabolic risk factors through multiple pathways.

Our article included sufficient studies that there was no publication bias (Egger’s test P>0.05) in almost all outcomes. Besides, we first reported the significant subgroup differences in T2DM and FBG in severe CCVD subgroup analysis, which could explain the controversial conclusion in the previously published articles. However, there were several limitations in our study: (1) Meta-regression is unavailable to subgroup data from less than ten studies, leading to a low R-squared of regression model. Therefore, our article only analyzed confounding factors through subgroup analysis rather than adjusting the effect of alcohol consumption or BMI on outcomes of cardiometabolic risk factors. (2) We did not conduct the subgroup analysis of drinker/non-drinker, male/female, and different genotypes (for example, GG *vs*. AA) due to the insufficient (less than five studies) subgroup data in the included studies. Even though we analyzed these subgroup analyses, the heterogeneity was too high to obtain a credible conclusion. (3) Although we believed that alcohol consumption, dietary habits, and BMI were the critical factors between ALDH2 and cardiometabolic risk factors, due to the lack of original data of included studies, we could not conduct the mediating analysis and interaction effects in our study. Future studies were needed to confirm the cardiometabolic mechanism of ALDH2.

## Conclusion

Our updated meta-analysis demonstrated that ALDH2 rs671 GG populations had significantly higher levels of BMI, blood pressure, FBG, TG, LDL-C and higher risk of hypertension than GA/AA populations. Besides, to the best of our knowledge, we first report GG had a higher risk of T2DM in population without severe CCVDs, and GA/AA had a higher risk of T2DM in population with severe CCVDs. Future studies were warranted to confirm the cardiometabolic mechanism of ALDH2.

## Data availability statement

The original contributions presented in the study are included in the article/**Supplementary Material**. Further inquiries can be directed to the corresponding author.

## Author contributions

RL: Methodology, Project administration, Software, Writing – original draft. MP: Data curation, Funding acquisition, Investigation, Supervision, Writing – original draft. JZ: Data curation, Visualization, Writing – review & editing. KQ: Formal Analysis, Software, Visualization, Writing – review & editing. TZ: Formal Analysis, Supervision, Writing – review & editing. LC: Conceptualization, Supervision, Writing – review & editing.

## Supplementary material

Supplementary Figure S1 Tetramer structure of ALDH2 enzyme

Supplementary Figure S2 Forest plot of ALDH2 GA/AA vs. GG on Age

Supplementary Figure S3 Forest plot of ALDH2 GA/AA vs. GG on Gender

Supplementary Figure S4 Forest plot of ALDH2 GA/AA vs. GG on Smoker

Supplementary Figure S5 Forest plot of ALDH2 GA/AA vs. GG on SBP

Supplementary Figure S6 Forest plot of ALDH2 GA/AA vs. GG on DBP

Supplementary Figure S7 Forest plot of ALDH2 GA/AA vs. GG on FBG

Supplementary Figure S8 Forest plot of ALDH2 GA/AA vs. GG on HbA1c

Supplementary Figure S9 Forest plot of ALDH2 GA/AA *vs*. GG on TC

Supplementary Figure S10 Forest plot of ALDH2 GA/AA *vs*. GG on TG

Supplementary Figure S11 Forest plot of ALDH2 GA/AA vs. GG on LDL-C

Supplementary Figure S12 Forest plot of ALDH2 GA/AA vs. GG on HDL-C

Supplementary Figure S13 Funnel plot of outcome of BMI

Supplementary Figure S14 Funnel plot of outcome of Hypertension

Supplementary Figure S15 Funnel plot of outcome of T2DM

Supplementary Table 1 PRISMA Checklist of our study
