# Supplementary material for: The ALDH2 gene rs671 polymorphism is associated with cardiometabolic risk factors in East Asian population: an updated meta-analysis
Source: Front Endocrinol (Lausanne). 2024 Mar 19;15:1333595. doi: 10.3389/fendo.2024.1333595 (PMC10986734; doi:10.3389/fendo.2024.1333595)
Supplement: Supplementary Figure S1 — Tetramer structure of ALDH2 enzyme [file DataSheet_1.zip › Figure S1.PDF]

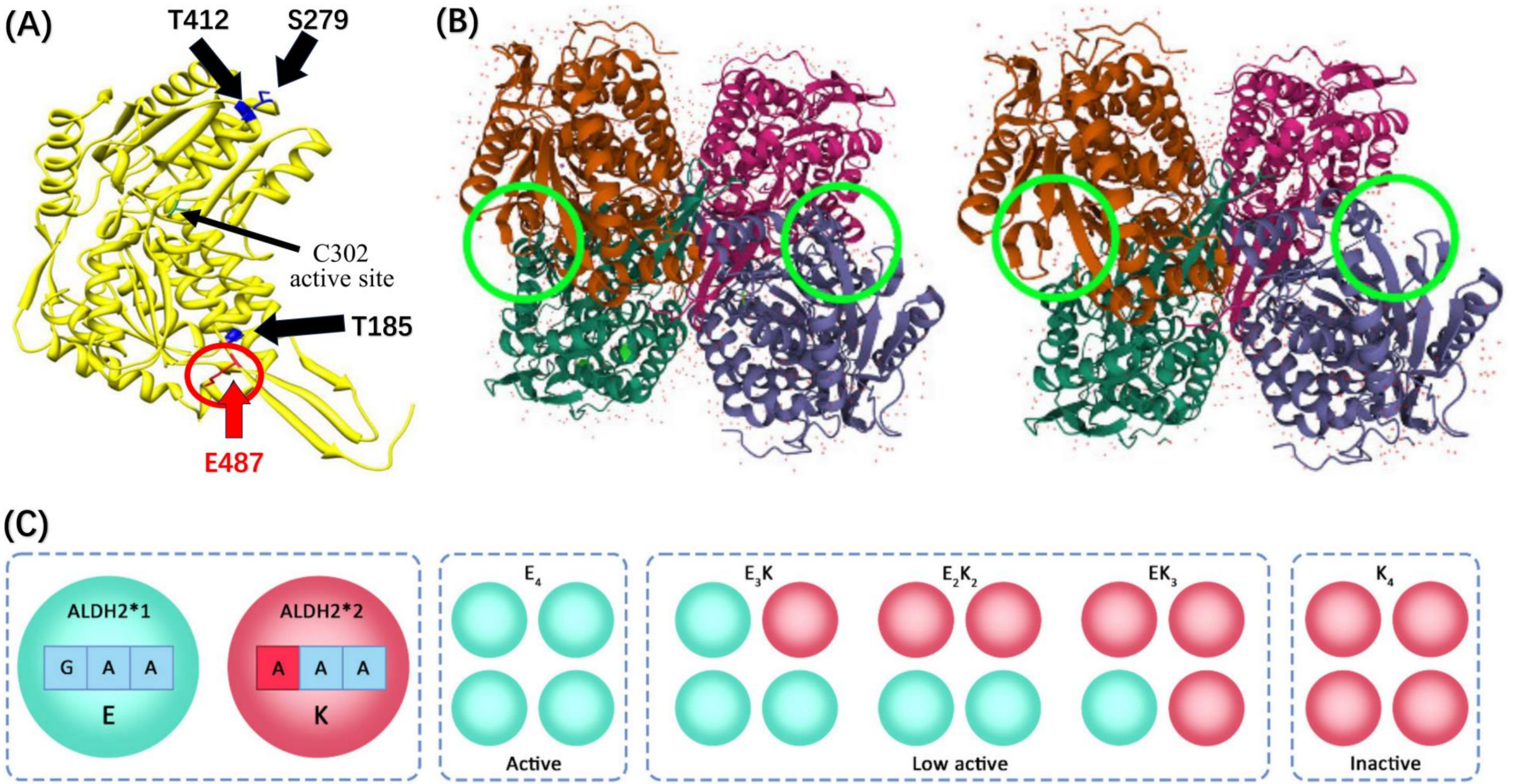

Figure S1. Tetramer structure of ALDH2 enzyme. A. ALDH2 monomer displaying the three phosphorylation sites: Thr185, Ser279, and Thr412 (blue). Also highlighted are the catalytic Cys302 (green) and the site of the ALDH2 mutation: Glu487 (red). B. Tetramer structure of wild-type ALDH2 (left) and inactive mutant of ALDH2. Green circle: the α-helix structure: missing from the mutant tetramer versus WT. C. "E" = subunit encoded by the wild-type allele ALDH2 G; the first base of codon 487 is guanine (G), and the 487th amino acid residue is glutamic acid (Glu/E). "K" = subunit encoded by the mutant allele ALDH2 A; the first base of codon 487 is mutated to adenine (A), and the 487th amino acid residue is changed from Glu to lysine (Lys/K). E4 homotetramers reflect normal enzyme activity; heterologous tetramer E3K, E2K2, and EK3 enzyme activities are substantially reduced; and K4 homotetramers enzyme activities are almost lost.

Reference: 1. Nene A, Chen CH, Disatnik MH, Cruz L, Mochly-Rosen D. Aldehyde dehydrogenase 2 activation and coevolution of its εPKC-mediated phosphorylation sites. *J Biomed Sci* (2017) 24(1):3. doi: 10.1186/s12929-016-0312-x. 2. Gao J, Hao Y, Piao X, Gu X. Aldehyde Dehydrogenase 2 as a Therapeutic Target in Oxidative Stress-Related Diseases: Post-Translational Modifications Deserve More Attention. *Int J Mol Sci* (2022) 23(5):2682. doi: 10.3390/ijms23052682.
