# Supplementary material for: The ALDH2 gene rs671 polymorphism is associated with cardiometabolic risk factors in East Asian population: an updated meta-analysis
Source: Front Endocrinol (Lausanne). 2024 Mar 19;15:1333595. doi: 10.3389/fendo.2024.1333595 (PMC10986734; doi:10.3389/fendo.2024.1333595)
Supplement: Supplementary Figure S1 — Tetramer structure of ALDH2 enzyme [file DataSheet_1.zip › Table S4.DOCX]

Table S4. Subgroup analysis between different qualities of included studies

| Outcomes | Subgroup of quality^a^ | No. of study | Participants | Statistical method | 95% CI | Subgroup difference |
| --- | --- | --- | --- | --- | --- | --- |
| BMI | High | 16 | 31152 | MD | -0.25 [-0.32, -0.18] | 0.60 |
|  | Low | 6 | 14303 | MD | -0.29 [-0.42, -0.16] |  |
| Hypertention | High | 25 | 48862 | OR | 0.83 [0.80, 0.86] | 0.49 |
|  | Low | 4 | 19584 | OR | 0.85 [0.80, 0.90] |  |
| SBP | High | 12 | 29849 | MD | -1.26 [-1.70, -0.83] | 0.11 |
|  | Low | 5 | 16899 | MD | -1.84 [-2.39, -1.28] |  |
| DBP | High | 12 | 29849 | MD | -0.74 [-1.15, -0.34] | 0.12 |
|  | Low | 5 | 16899 | MD | -1.80 [-3.07, -0.53] |  |
| T2DM | High | 15 | 32191 | OR | 1.01 [0.86, 1.18] | 0.80 |
|  | Low | 5 | 19238 | OR | 1.05 [0.79, 1.40] |  |
| FBG | High | 14 | 21144 | MD | -0.10 [-0.13, -0.06] | 0.94 |
|  | Low | 5 | 14010 | MD | -0.10 [-0.14, -0.05] |  |
| HbA1c | High | 5 | 5113 | MD | 0.02 [-0.03, 0.06] | 0.26 |
|  | Low | 5 | 1522 | MD | 0.08 [-0.02, 0.18] |  |
| TC | High | 11 | 19370 | MD | -0.02 [-0.05, 0.01] | 0.82 |
|  | Low | 5 | 17614 | MD | -0.02 [-0.05, 0.01] |  |
| TG | High | 15 | 25805 | MD | -0.08 [-0.12, -0.04] | 0.42 |
|  | Low | 5 | 17614 | MD | -0.06 [-0.07, -0.05] |  |
| LDL-C | High | 13 | 20723 | MD | -0.03 [-0.05, -0.01] | 0.54 |
|  | Low | 4 | 17238 | MD | -0.04 [-0.07, -0.02] |  |
| HDL-C | High | 14 | 25123 | MD | -0.01 [-0.05, 0.03] | 0.53 |
|  | Low | 5 | 17614 | MD | -0.02 [-0.05, 0.00] |  |

^a^Low quality was defined as: (1) P(HWE)≤0.05; (2) NOS ≤5 point; (3) AHRQ ≤7 point.
