# Supplementary material for: The ALDH2 gene rs671 polymorphism is associated with cardiometabolic risk factors in East Asian population: an updated meta-analysis
Source: Front Endocrinol (Lausanne). 2024 Mar 19;15:1333595. doi: 10.3389/fendo.2024.1333595 (PMC10986734; doi:10.3389/fendo.2024.1333595)
Supplement: Supplementary Figure S1 — Tetramer structure of ALDH2 enzyme [file DataSheet_1.zip › Table S5.DOCX]

Table S5. Subgroup analysis between different groups of nationality

| Outcomes | Nationality^a^ | No. of study | Participants | Statistical method | 95% CI | Subgroup difference |
| --- | --- | --- | --- | --- | --- | --- |
| BMI | Chinese | 8 | 25509 | MD | -0.28 [-0.37, -0.19] | 0.90 |
|  | Japanese | 14 | 11420 | MD | -0.29 [-0.41, -0.17] |  |
| hyp | Chinese | 19 | 38768 | OR | 0.84 [0.80, 0.87] | 0.20 |
|  | Japanese | 9 | 21152 | OR | 0.80 [0.75, 0.85] |  |
| SBP | Chinese | 7 | 29360 | MD | -1.87 [-2.83, -0.92] | 0.19 |
|  | Japanese | 10 | 8862 | MD | -0.97 [-1.91, -0.03] |  |
| DBP | Chinese | 7 | 29360 | MD | -1.25 [-2.01, -0.49] | 0.52 |
|  | Japanese | 10 | 8862 | MD | -0.90 [-1.67, -0.12] |  |
| T2DM | Chinese | 12 | 26909 | OR | 1.09 [0.94, 1.27] | 0.44 |
|  | Japanese | 7 | 15994 | OR | 0.93 [0.65, 1.34] |  |
| FPG | Chinese | 6 | 25632 | MD | -0.06 [-0.16, 0.04] | 0.60 |
|  | Japanese | 13 | 9522 | MD | -0.09 [-0.14, -0.04] |  |
| HbA1c | Chinese | 1 | 613 | MD | 0.10 [-0.06, 0.26] | 0.36 |
|  | Japanese | 9 | 6022 | MD | 0.02 [-0.02, 0.06] |  |
| TC | Chinese | 12 | 31191 | MD | -0.03 [-0.05, -0.01] | 0.13 |
|  | Japanese | 4 | 5793 | MD | 0.01 [-0.03, 0.05] |  |
| TG | Chinese | 13 | 35486 | MD | -0.07 [-0.10, -0.04] | 0.68 |
|  | Japanese | 7 | 7933 | MD | -0.08 [-0.16, -0.01] |  |
| LDL-C | Chinese | 13 | 35486 | MD | -0.04 [-0.05, -0.02] | 0.35 |
|  | Japanese | 4 | 2475 | MD | -0.01 [-0.07, 0.05] |  |
| HDL-C | Chinese | 12 | 33367 | MD | -0.02 [-0.03, -0.00] | 0.95 |
|  | Japanese | 6 | 7251 | MD | -0.02 [-0.16, 0.11] |  |
| ^a^Data of Korean only included in one study, so the Korean population was excluded in this subgroup analysis. | | | | | | |
