# Supplementary material for: The ALDH2 gene rs671 polymorphism is associated with cardiometabolic risk factors in East Asian population: an updated meta-analysis
Source: Front Endocrinol (Lausanne). 2024 Mar 19;15:1333595. doi: 10.3389/fendo.2024.1333595 (PMC10986734; doi:10.3389/fendo.2024.1333595)
Supplement: Supplementary Figure S1 — Tetramer structure of ALDH2 enzyme [file DataSheet_1.zip › Table S6.DOCX]

Table S6. Subgroup analysis between different article types

| Outcomes | Article type | No. of study | Participants | Statistical method | 95% CI | Subgroup difference |
| --- | --- | --- | --- | --- | --- | --- |
| BMI | Cross-sectional | 10 | 24133 | MD | -0.27 [-0.37, -0.18] | 0.85 |
|  | Cohort | 7 | 9696 | MD | -0.23 [-0.35, -0.11] |  |
|  | Case-control | 5 | 11626 | MD | -0.26 [-0.39, -0.14] |  |
| Hypertention | Cross-sectional | 11 | 38775 | OR | 0.79 [0.75, 0.84] | 0.09 |
|  | Cohort | 14 | 18145 | OR | 0.87 [0.78, 0.98] |  |
|  | Case-control | 4 | 11526 | OR | 0.88 [0.81, 0.95] |  |
| SBP | Cross-sectional | 6 | 20929 | MD | -1.31 [-1.78, -0.83] | 0.49 |
|  | Cohort | 5 | 9326 | MD | -1.53 [-2.26, -0.79] |  |
|  | Case-control | 6 | 16493 | MD | -1.77 [-2.43, -1.12] |  |
| DBP | Cross-sectional | 6 | 20929 | MD | -0.93 [-1.84, -0.02] | 0.58 |
|  | Cohort | 5 | 9326 | MD | -1.05 [-1.50, -0.60] |  |
|  | Case-control | 6 | 16493 | MD | -1.22 [-2.15, -0.29] |  |
| T2DM | Cross-sectional | 8 | 28058 | OR | 0.92 [0.76, 1.13] | 0.09 |
|  | Cohort | 7 | 18245 | OR | 0.95 [0.72, 1.26] |  |
|  | Case-control | 5 | 5126 | OR | 1.24 [1.02, 1.50] |  |
| FBG | Cross-sectional | 8 | 25141 | MD | -0.10 [-0.13, -0.07] | 0.19 |
|  | Cohort | 8 | 3507 | MD | -0.15 [-0.19, -0.11] |  |
|  | Case-control | 3 | 4918 | MD | 0.01 [-0.29, 0.32] |  |
| HbA1c | Cross-sectional | 4 | 5460 | MD | 0.02 [-0.03, 0.07] | 0.33 |
|  | Cohort | 4 | 627 | MD | 0.01 [-0.07, 0.08] |  |
|  | Case-control | 2 | 548 | MD | 0.10 [-0.01, 0.22] |  |
| TC | Cross-sectional | 9 | 24223 | MD | -0.01 [-0.03, 0.01] | 0.19 |
|  | Cohort | 1 | 159 | MD | 0.26 [-0.13, 0.65] |  |
|  | Case-control | 6 | 12602 | MD | -0.04 [-0.08, -0.01] |  |
| TG | Cross-sectional | 9 | 25694 | MD | -0.08 [-0.12, -0.03] | 0.16 |
|  | Cohort | 3 | 594 | MD | -0.14 [-0.31, 0.03] |  |
|  | Case-control | 8 | 17131 | MD | -0.04 [-0.07, -0.02] |  |
| LDL-C | Cross-sectional | 6 | 20236 | MD | -0.03 [-0.05, -0.00] | 0.38 |
|  | Cohort | 3 | 594 | MD | 0.01 [-0.11, 0.13] |  |
|  | Case-control | 8 | 17131 | MD | -0.05 [-0.08, -0.02] |  |
| HDL-C | Cross-sectional | 8 | 25012 | MD | -0.01 [-0.06, 0.04] | 0.78 |
|  | Cohort | 2 | 435 | MD | -0.04 [-0.12, 0.04] |  |
|  | Case-control | 9 | 17290 | MD | -0.02 [-0.04, 0.01] |  |
| *P≤0.05 | | | | | | |
