# Supplementary material for: The ALDH2 gene rs671 polymorphism is associated with cardiometabolic risk factors in East Asian population: an updated meta-analysis
Source: Front Endocrinol (Lausanne). 2024 Mar 19;15:1333595. doi: 10.3389/fendo.2024.1333595 (PMC10986734; doi:10.3389/fendo.2024.1333595)
Supplement: Supplementary Figure S1 — Tetramer structure of ALDH2 enzyme [file DataSheet_1.zip › Table S7.DOCX]

Table S7. Subgroup analysis between studies with and without severe CCVD populations

| Outcomes | Severe CCVDs^a^ | No. of study | Participant | Statistical method | 95% CI | Subgroup difference | |
| --- | --- | --- | --- | --- | --- | --- | --- |
| BMI | With | 2 | 511 | MD | -0.37 [-1.03, 0.29] | 0.73 | |
|  | Without | 20 | 44944 | MD | -0.26 [-0.32, -0.19] |  | |
| T2DM | With | 9 | 5510 | OR | 1.29 [1.00, 1.66] | 0.006* | |
|  | Without | 11 | 45919 | OR | 0.88 [0.79, 0.97] |  | |
| FBG | With | 3 | 686 | MD | 0.25 [0.09, 0.41] | <0.001* | |
|  | Without | 16 | 34468 | MD | -0.11 [-0.14, -0.08] |  | |
| Hypertention | With | 6 | 1758 | OR | 0.90 [0.73, 1.12] | 0.38 | |
|  | Without | 23 | 66688 | OR | 0.81 [0.77, 0.87] |  | |
| TC | With | 4 | 1028 | MD | 0.01 [-0.15, 0.17] | 0.70 | |
|  | Without | 12 | 35956 | MD | -0.02 [-0.04, 0.00] |  | |
| TG | With | 4 | 1028 | MD | -0.05 [-0.19, 0.08] | 0.83 | |
|  | Without | 16 | 42391 | MD | -0.07 [-0.10, -0.04] |  | |
| LDL-C | With | 4 | 1028 | MD | 0.00 [-0.11, 0.12] | 0.50 | |
|  | Without | 13 | 36933 | MD | -0.04 [-0.05, -0.02] |  | |
| HDL-C | With | 4 | 1028 | MD | -0.02 [-0.07, 0.03] | 0.86 | |
|  | Without | 15 | 41709 | MD | -0.01 [-0.04, 0.01] |  | |
| Abbreviation: CCVDs=cardio-cerebral vascular disease. ^a^Severe CCVDs included myocardial infarct, coronary artery disease, ischemic stroke and hemorrhagic stroke. *P≤0.05 | | | | | | |  |
