# Supplementary figures and images for: The ALDH2 gene rs671 polymorphism is associated with cardiometabolic risk factors in East Asian population: an updated meta-analysis
Source: Front Endocrinol (Lausanne). 2024 Mar 19;15:1333595. doi: 10.3389/fendo.2024.1333595 (PMC10986734; doi:10.3389/fendo.2024.1333595)

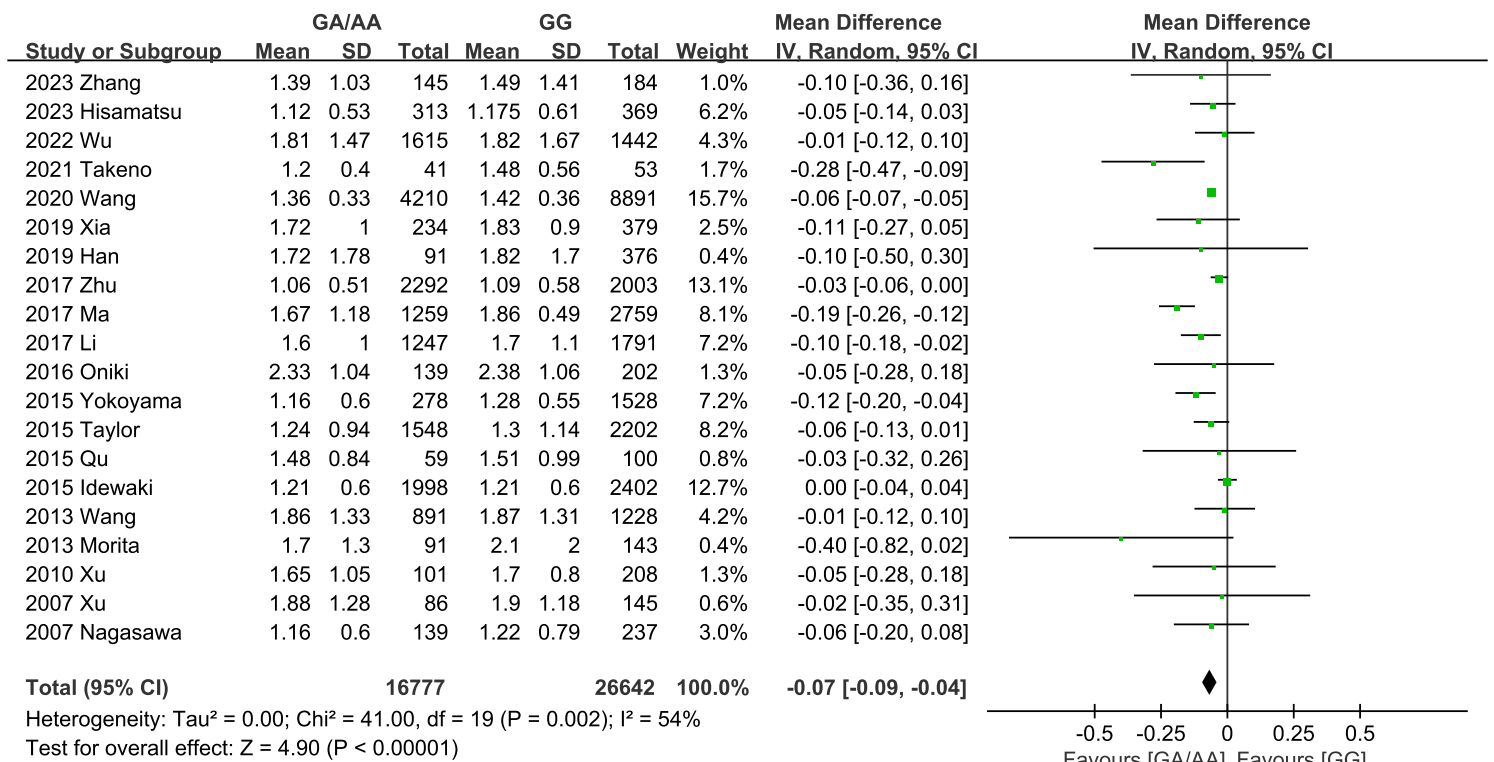

Supplement: Supplementary Figure S1 — Tetramer structure of ALDH2 enzyme [file DataSheet_1.zip › Figure S10.PDF]

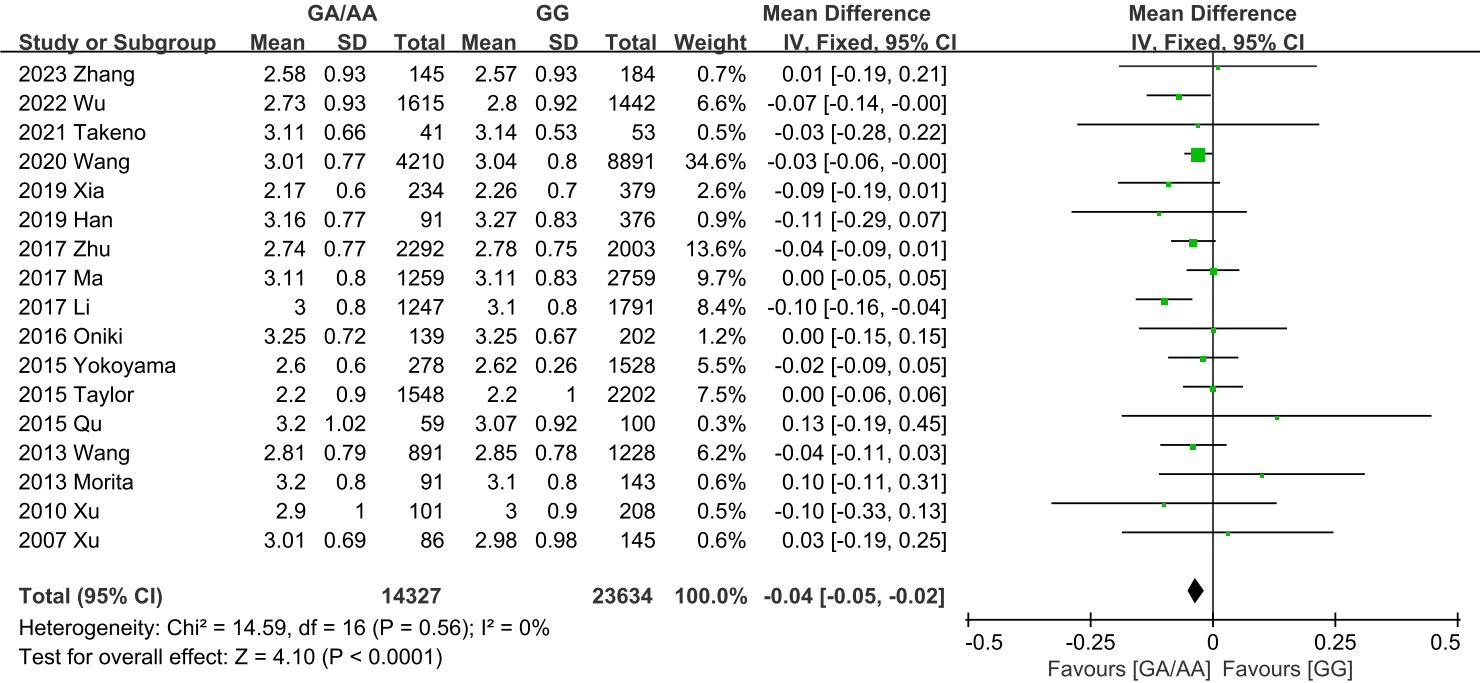

Supplement: Supplementary Figure S1 — Tetramer structure of ALDH2 enzyme [file DataSheet_1.zip › Figure S11.PDF]

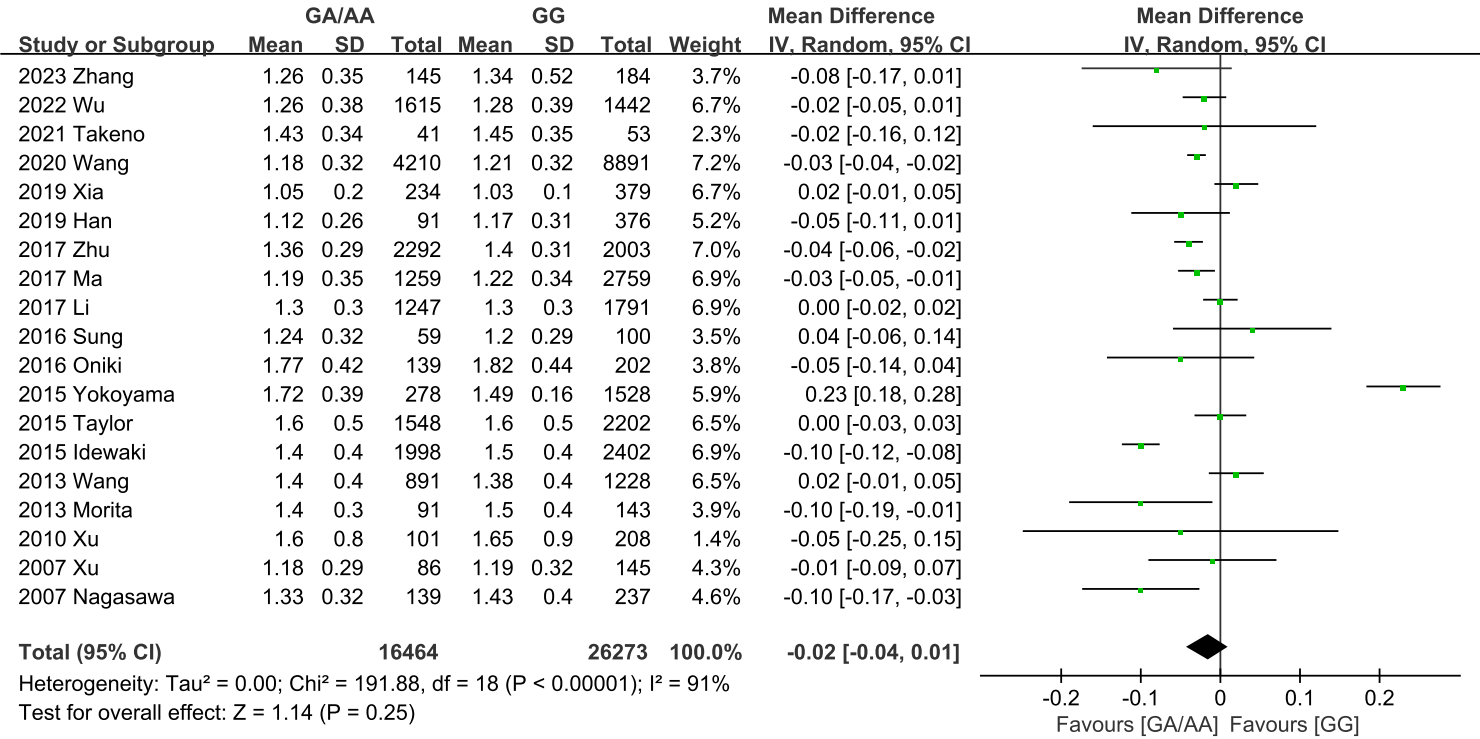

Supplement: Supplementary Figure S1 — Tetramer structure of ALDH2 enzyme [file DataSheet_1.zip › Figure S12.PDF]

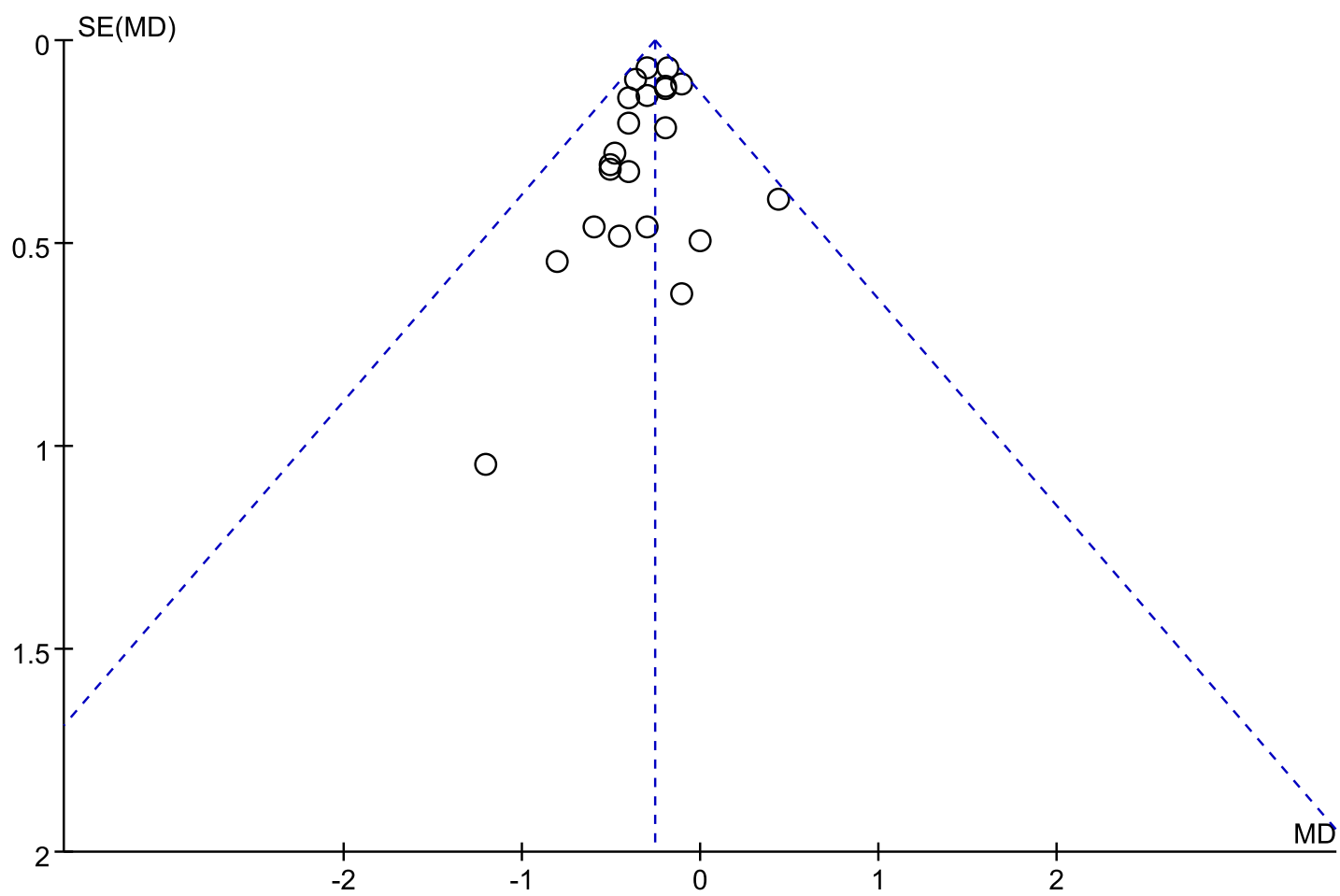

Supplement: Supplementary Figure S1 — Tetramer structure of ALDH2 enzyme [file DataSheet_1.zip › Figure S13.PDF]

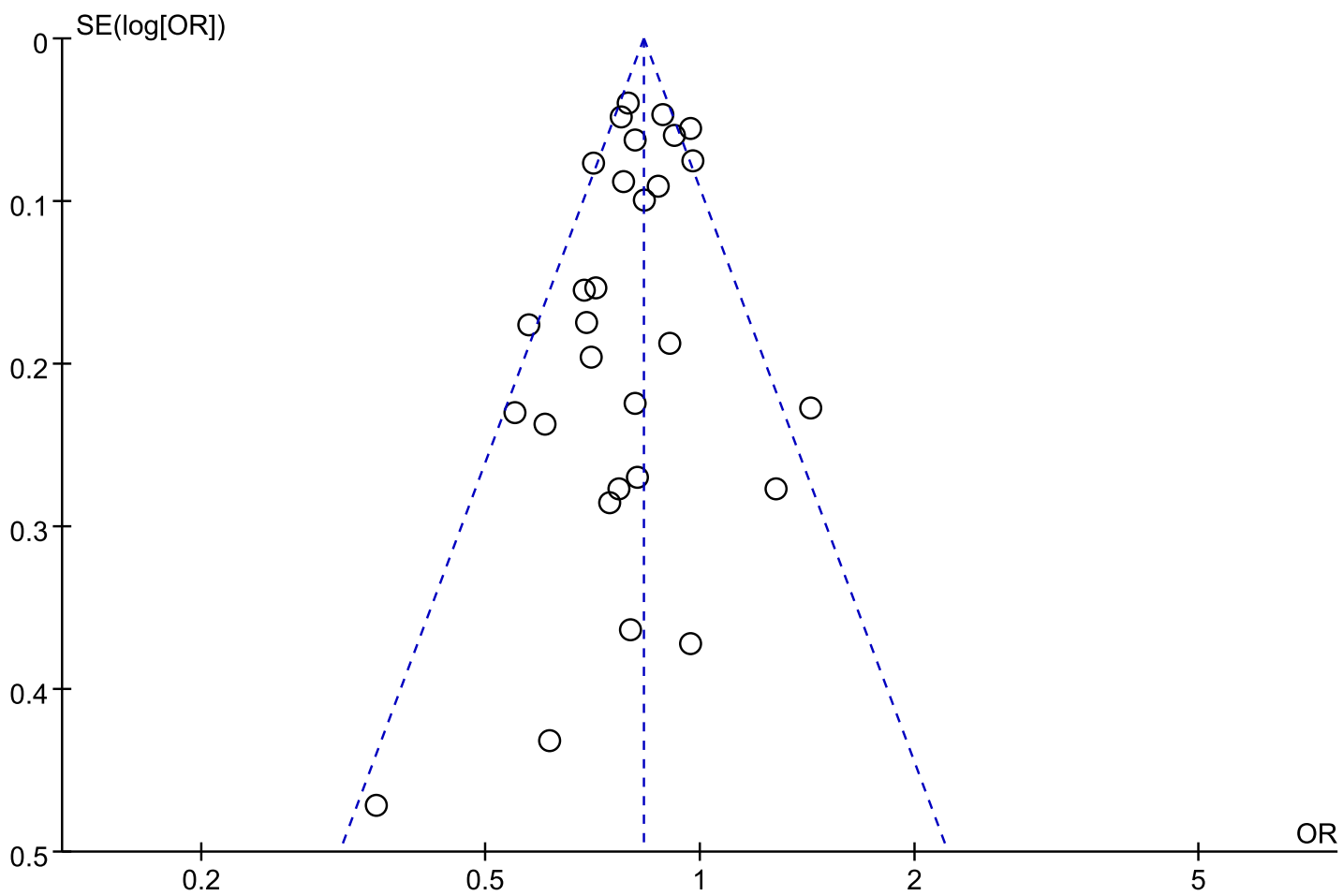

Supplement: Supplementary Figure S1 — Tetramer structure of ALDH2 enzyme [file DataSheet_1.zip › Figure S14.PDF]

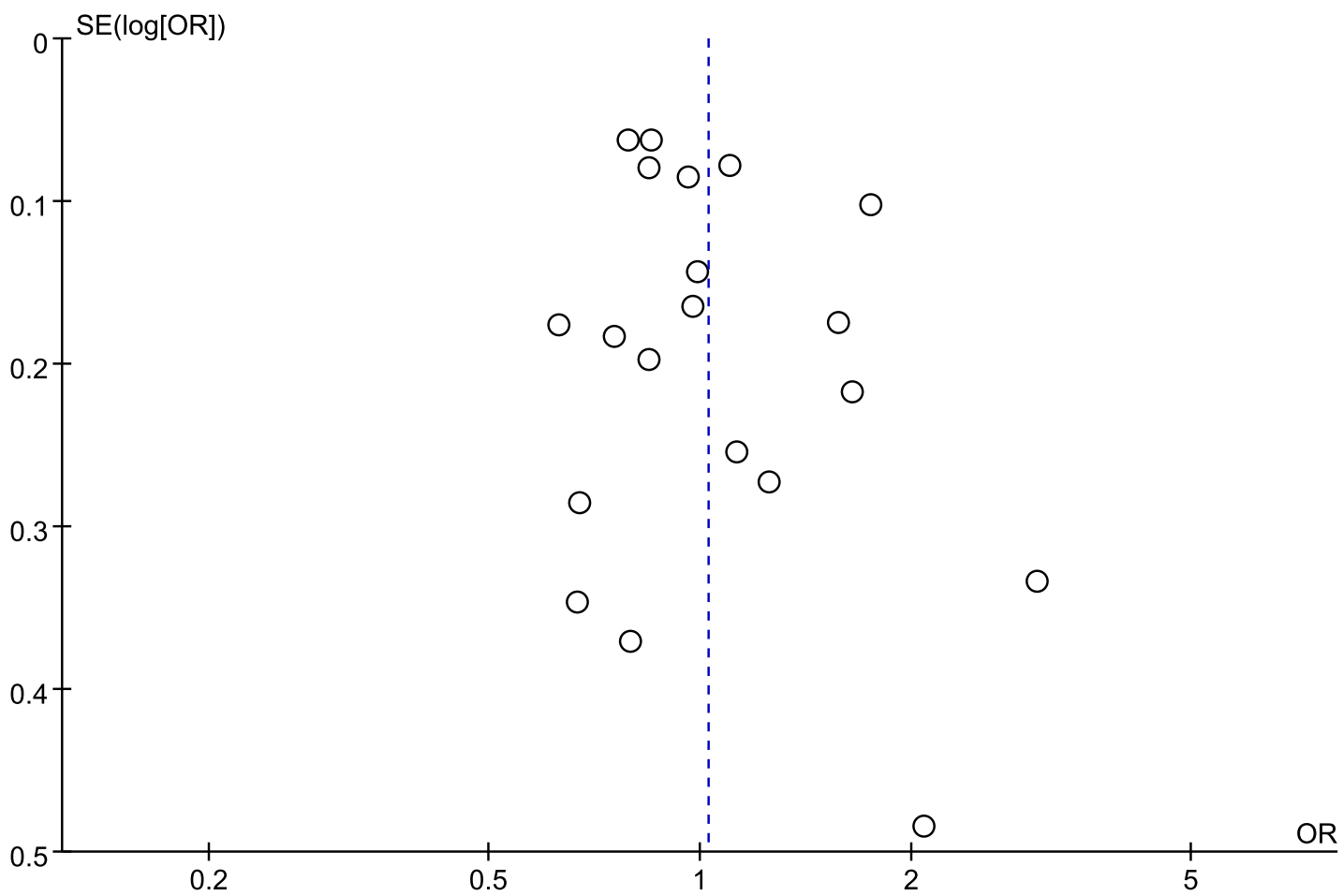

Supplement: Supplementary Figure S1 — Tetramer structure of ALDH2 enzyme [file DataSheet_1.zip › Figure S15.PDF]

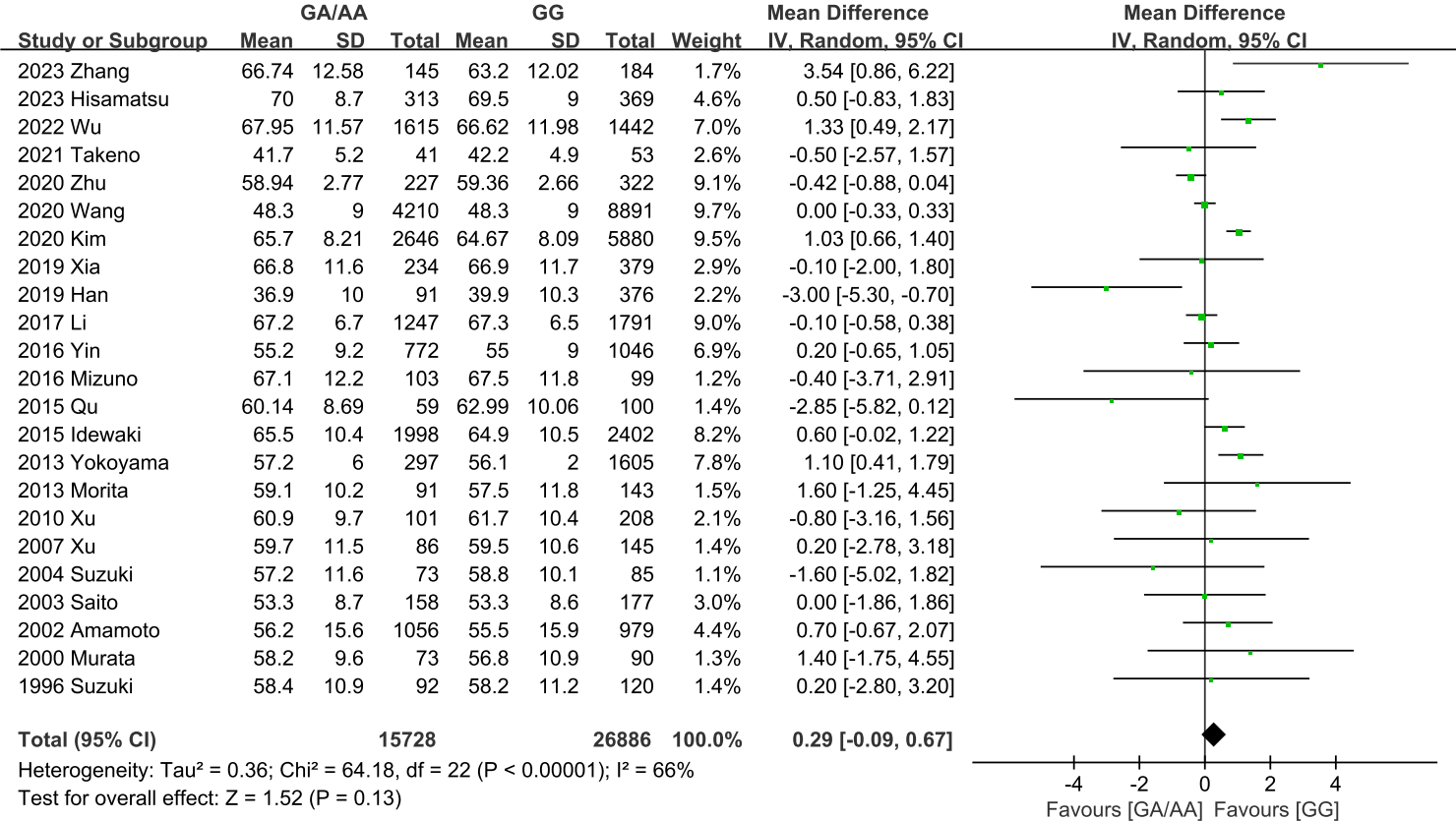

Supplement: Supplementary Figure S1 — Tetramer structure of ALDH2 enzyme [file DataSheet_1.zip › Figure S2.PDF]

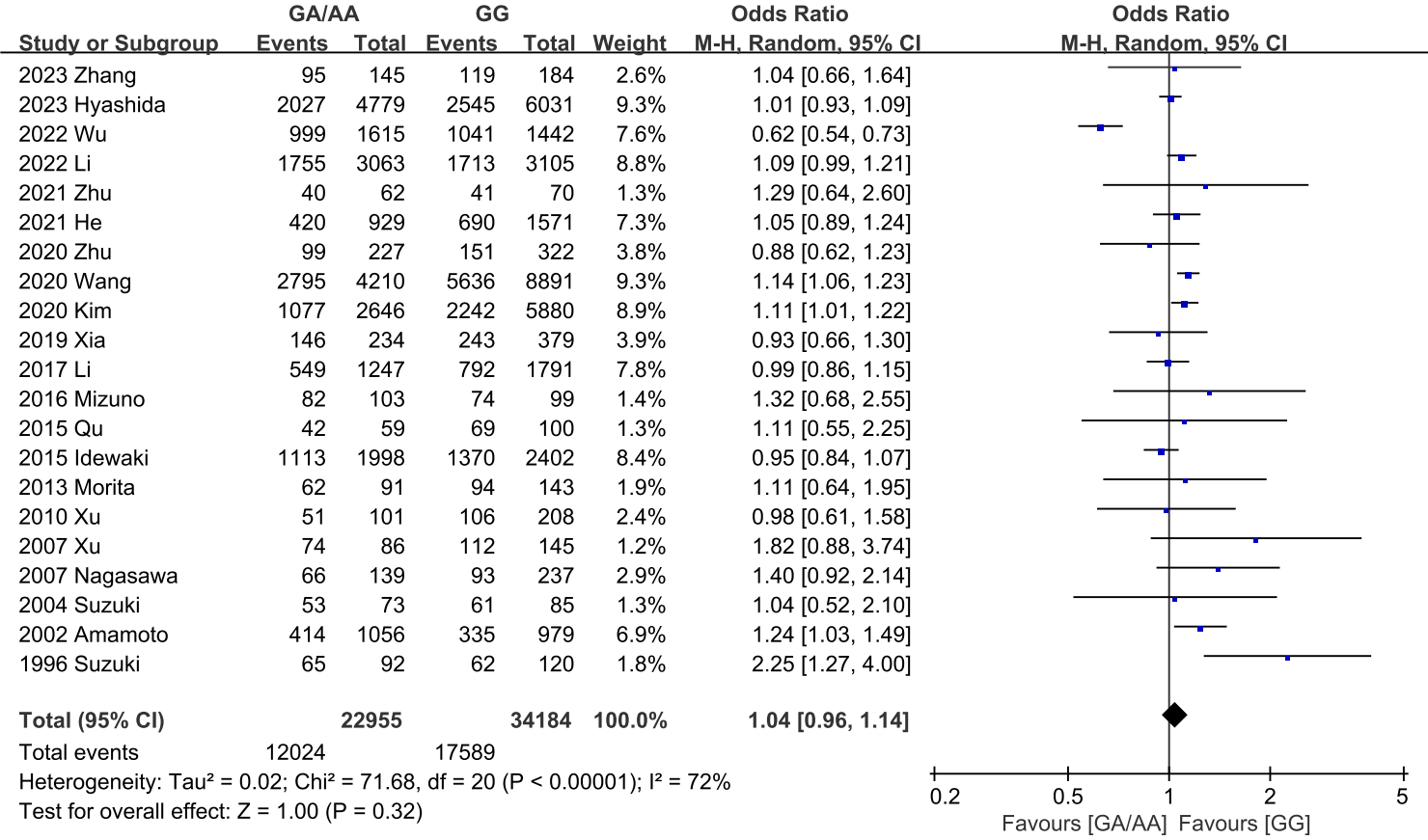

Supplement: Supplementary Figure S1 — Tetramer structure of ALDH2 enzyme [file DataSheet_1.zip › Figure S3.PDF]

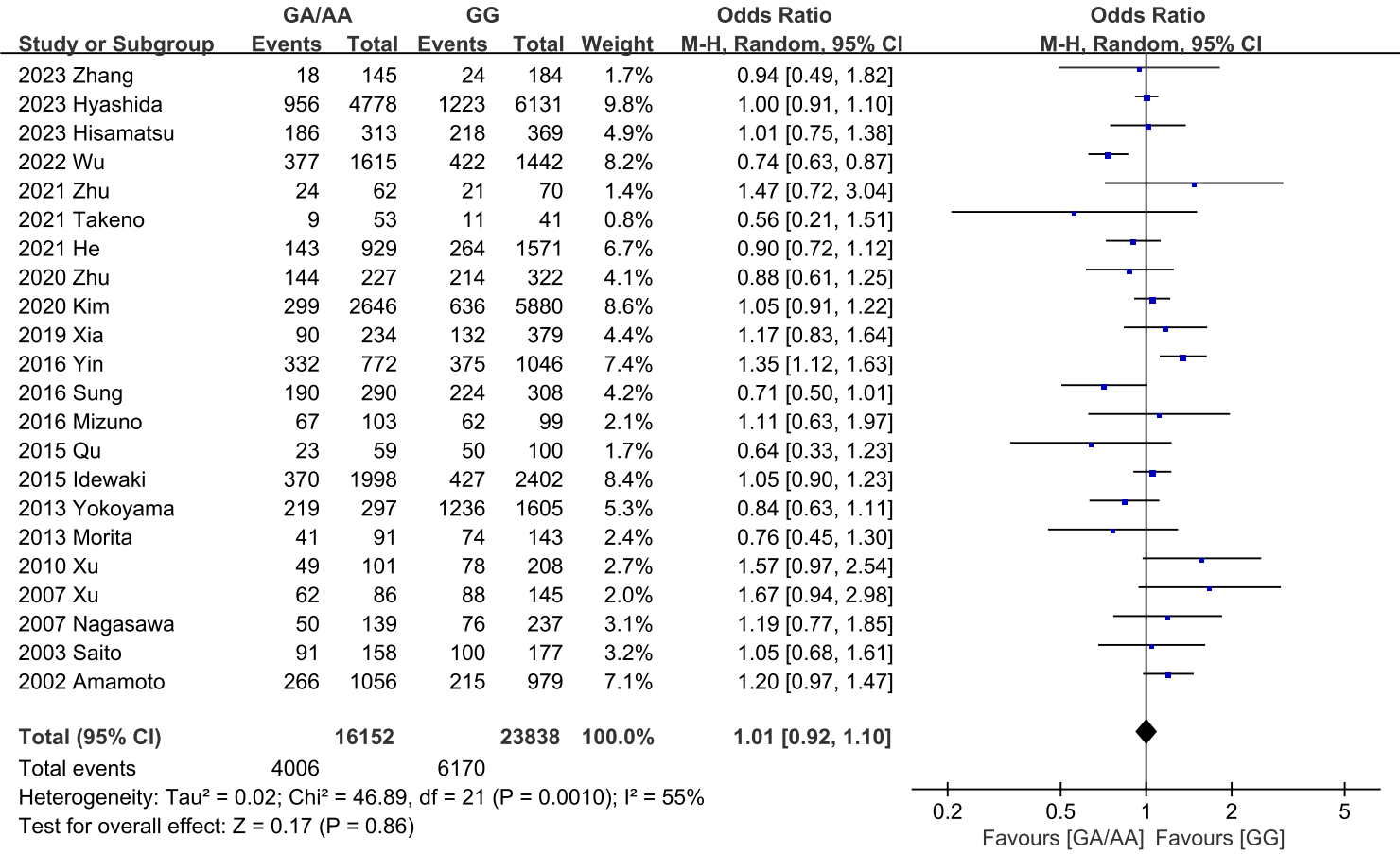

Supplement: Supplementary Figure S1 — Tetramer structure of ALDH2 enzyme [file DataSheet_1.zip › Figure S4.PDF]

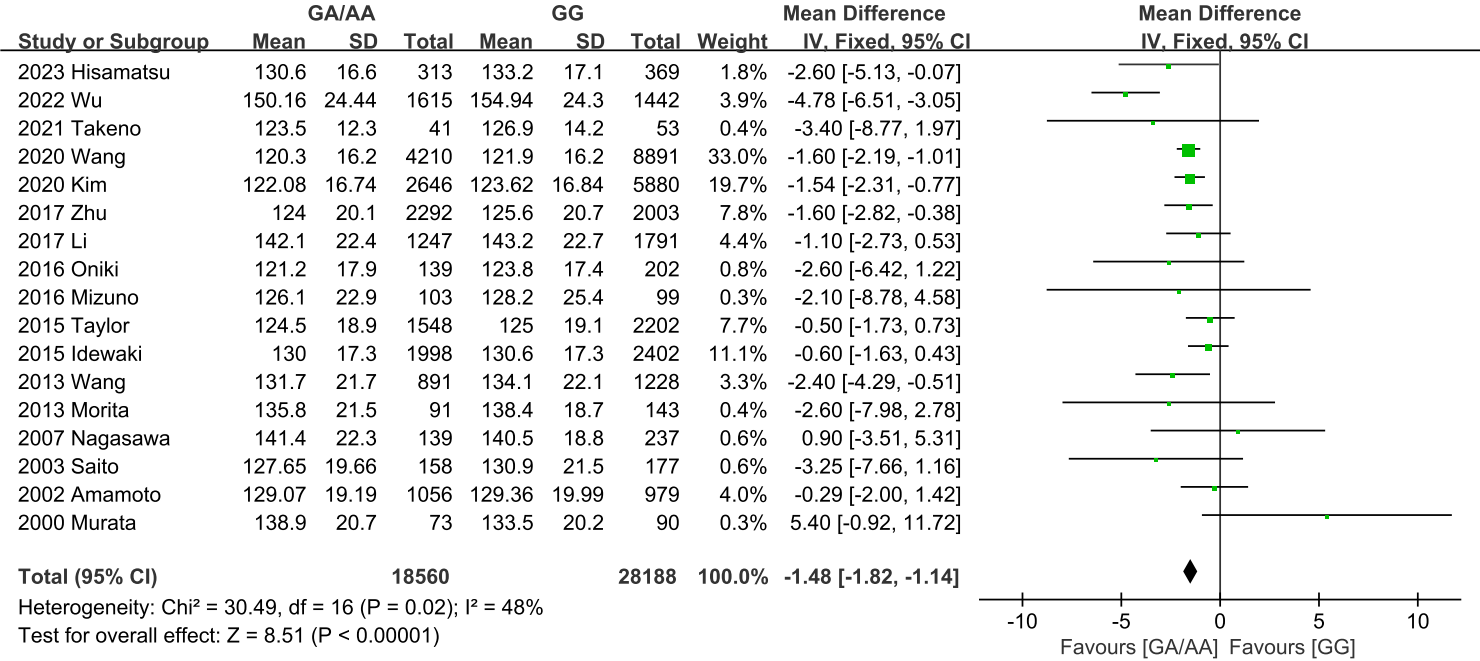

Supplement: Supplementary Figure S1 — Tetramer structure of ALDH2 enzyme [file DataSheet_1.zip › Figure S5.PDF]

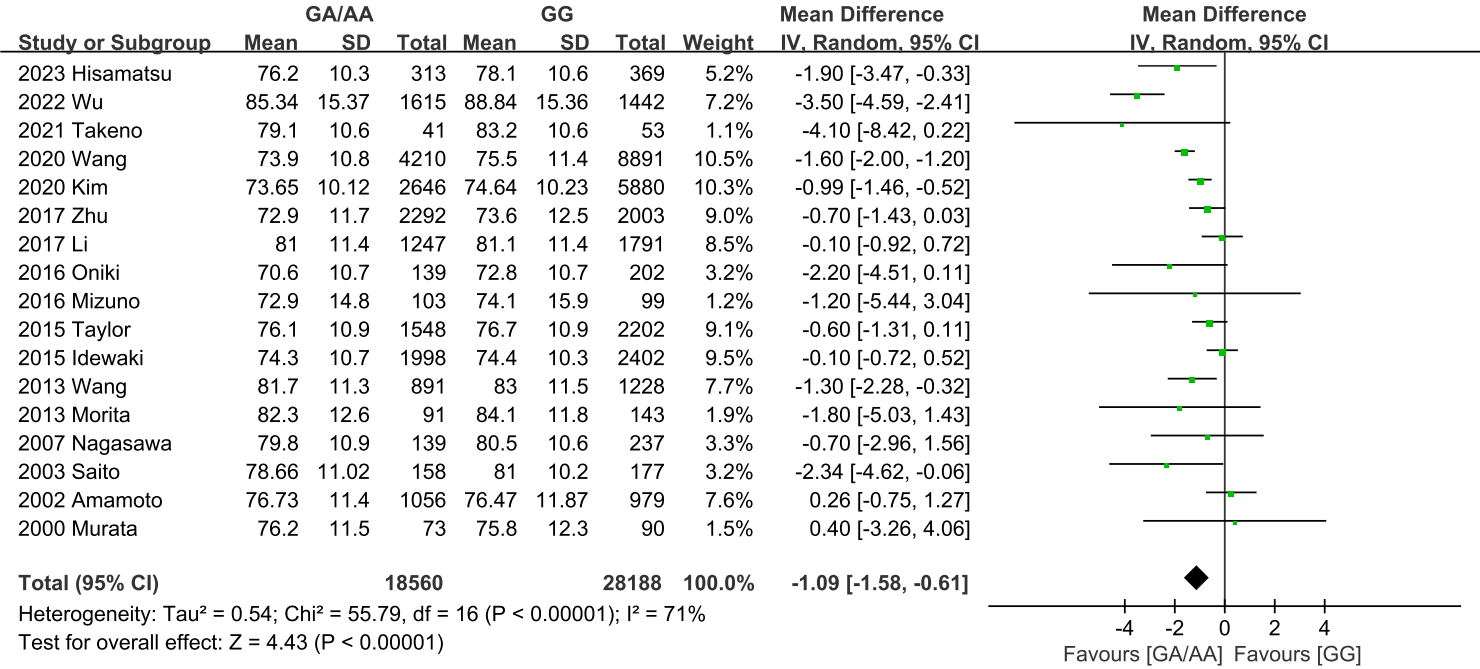

Supplement: Supplementary Figure S1 — Tetramer structure of ALDH2 enzyme [file DataSheet_1.zip › Figure S6.PDF]

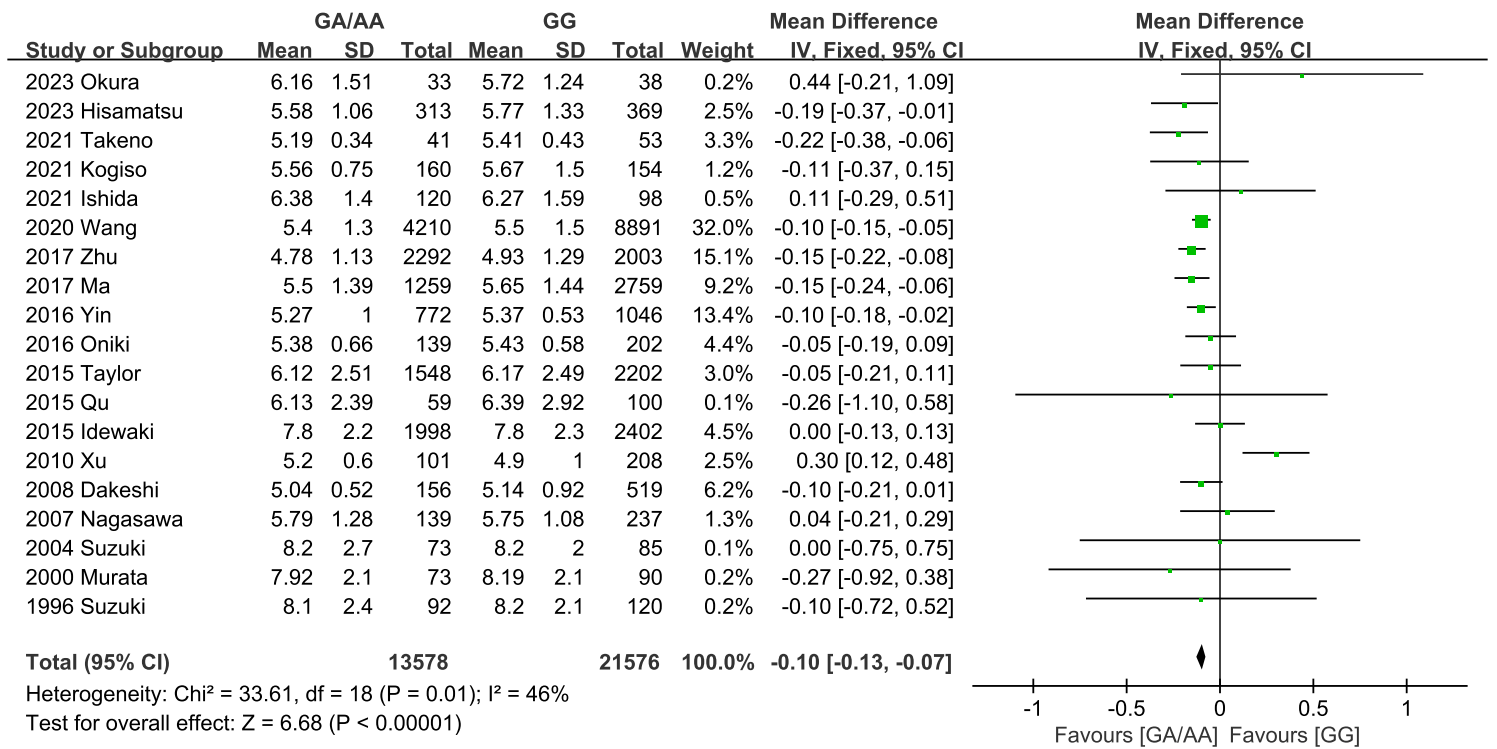

Supplement: Supplementary Figure S1 — Tetramer structure of ALDH2 enzyme [file DataSheet_1.zip › Figure S7.PDF]

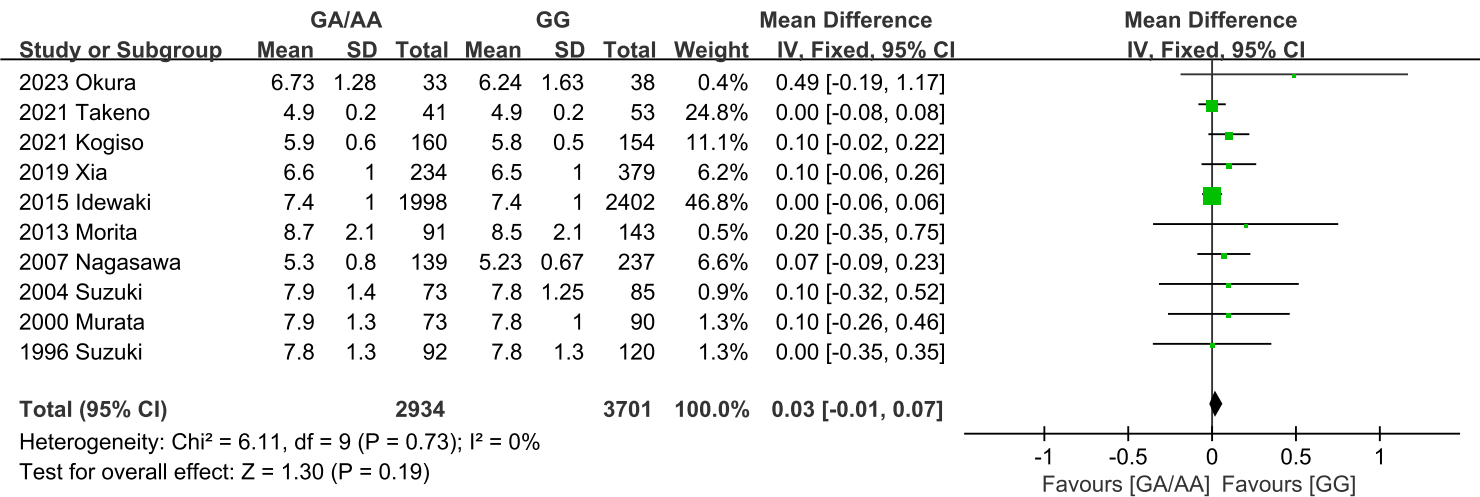

Supplement: Supplementary Figure S1 — Tetramer structure of ALDH2 enzyme [file DataSheet_1.zip › Figure S8.PDF]

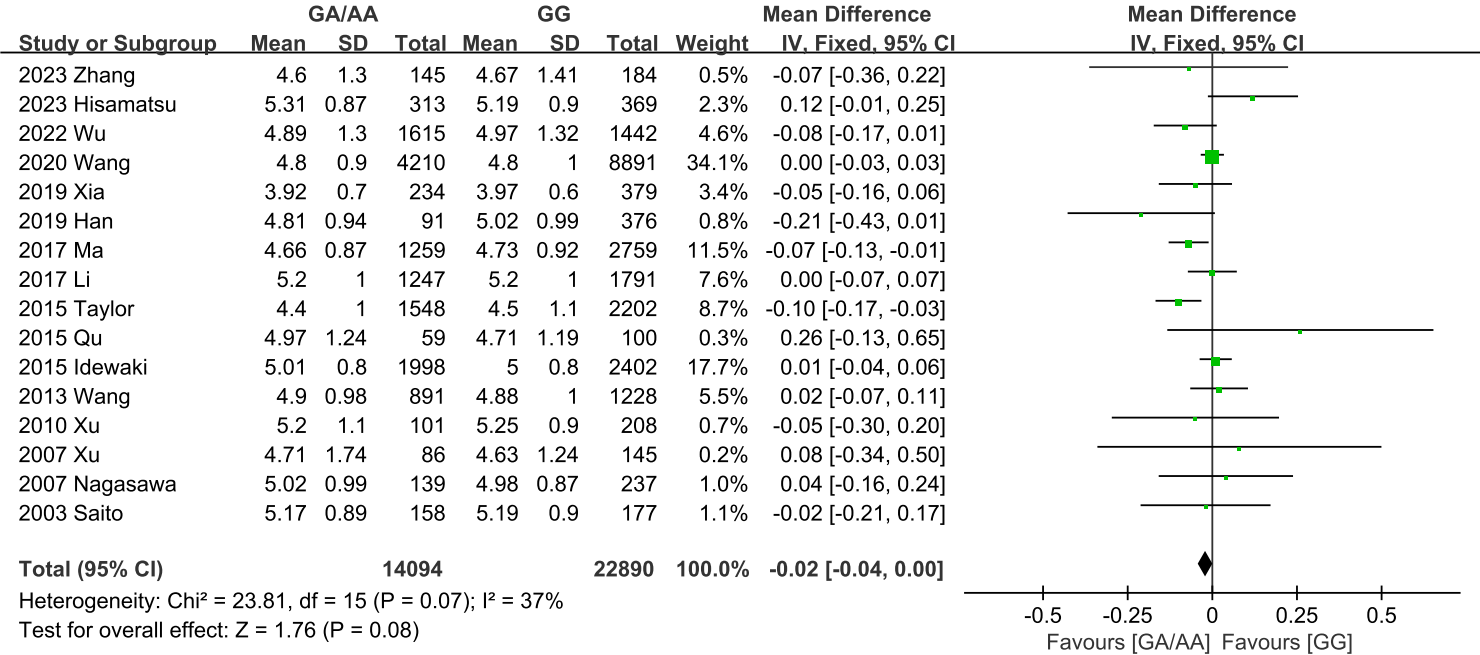

Supplement: Supplementary Figure S1 — Tetramer structure of ALDH2 enzyme [file DataSheet_1.zip › Figure S9.PDF]
