# Supplementary material for: The ALDH2 gene rs671 polymorphism is associated with cardiometabolic risk factors in East Asian population: an updated meta-analysis
Source: Front Endocrinol (Lausanne). 2024 Mar 19;15:1333595. doi: 10.3389/fendo.2024.1333595 (PMC10986734; doi:10.3389/fendo.2024.1333595)
Supplement: Supplementary Figure S1 — Tetramer structure of ALDH2 enzyme [file DataSheet_1.zip › Table S2.DOC]

Table S2. Search Strategy in Pubmed database

| Concept | Strategy | Result^*^ |
| --- | --- | --- |
| 1 | ALDH2*.ti.ab. | 2,602 |
| 2 | (polymorphism* OR SNP* OR Variant* OR Mutation*).ti.ab. | 1,886,021 |
| 3 | 1 AND 2 | 1,379 |
| 4 | (diabetes* OR obesity* or fat* or overweight*).ti.ab. | 1,511,160 |
| 5 | (Hypertension* OR blood pressure* OR SBP* OR DBP*) | 1,111,980 |
| 6 | (total cholesterol* OR triglyceride* OR LDL* OR HDL*) | 303,862 |
| 7 | (cardiovascular risk factor* OR cardiovascular disease*) | 2,981,953 |
| 8 | 4 OR 5 OR 6 OR 7 | 4,721,682 |
| 9 | 3 AND 8 | 306 |

^*^Updated to Oct 30^th^, 2023.

^#^ALDH2[Title/Abstract] AND (polymorphism OR SNP OR Variant OR Mutation OR rs671)[Title/Abstract] AND ((diabetes OR obesity or fat or overweight)[Title/Abstract] OR (Hypertension OR blood pressure OR SBP OR DBP)[Title/Abstract] OR (total cholesterol OR triglyceride OR LDL OR HDL)[Title/Abstract] OR (cardiovascular risk factor OR cardiovascular disease)[Title/Abstract])
