# Supplementary material for: The ALDH2 gene rs671 polymorphism is associated with cardiometabolic risk factors in East Asian population: an updated meta-analysis
Source: Front Endocrinol (Lausanne). 2024 Mar 19;15:1333595. doi: 10.3389/fendo.2024.1333595 (PMC10986734; doi:10.3389/fendo.2024.1333595)
Supplement: Supplementary Figure S1 — Tetramer structure of ALDH2 enzyme [file DataSheet_1.zip › Table S3.DOC]

Table S3.1. Assessment outcomes of included case-control studies

| NOS for Case-control study | Selection | | | | Comparability | | Exposure | | | Total score |
| --- | --- | --- | --- | --- | --- | --- | --- | --- | --- | --- |
|  | Case definition | Case represent-  ativeness | Control selection | Control definition | Controls for alcohol/  drinker | Controls for other factors | Ascertainment of exposure | Same ascertainment for control | Non-Response rate |  |
| Lan 2023 | 1 | 1 | 1 | 1 | 1 | 1 | 1 | 1 | 1 | 9 |
| Wu 2023 | 1 | 1 | 1 | 1 | 1 | 1 | 1 | 1 | 1 | 9 |
| Zhang 2023 | 1 | 0 | 1 | 1 | 0 | 1 | 1 | 1 | 1 | 7 |
| Kogiso 2021 | 1 | 0 | 1 | 1 | 0 | 0 | 1 | 1 | 1 | 6 |
| Zhu 2021 | 1 | 0 | 0 | 1 | 1 | 1 | 1 | 1 | 1 | 7 |
| Li 2017 | 1 | 1 | 1 | 1 | 0 | 1 | 1 | 1 | 1 | 8 |
| Zhu 2017 | 1 | 1 | 1 | 1 | 1 | 1 | 1 | 1 | 1 | 9 |
| Sung 2016 | 1 | 0 | 0 | 1 | 1 | 1 | 1 | 1 | 1 | 7 |
| Taylor 2015 | 1 | 1 | 1 | 1 | 0 | 1 | 1 | 1 | 1 | 8 |
| Lv 2013 | 1 | 1 | 1 | 1 | 0 | 0 | 1 | 1 | 1 | 7 |
| Morita 2013 | 1 | 0 | 1 | 1 | 0 | 1 | 1 | 1 | 1 | 7 |
| Wang 2013 | 1 | 0 | 1 | 1 | 1 | 1 | 1 | 1 | 1 | 8 |
| Feng 2012 | 1 | 1 | 1 | 1 | 0 | 0 | 1 | 1 | 1 | 7 |
| Hasi 2011 | 1 | 1 | 1 | 1 | 0 | 0 | 1 | 1 | 1 | 7 |
| Xu 2010 | 1 | 0 | 1 | 1 | 0 | 1 | 1 | 1 | 1 | 7 |
| Hui 2010 | 1 | 1 | 1 | 1 | 0 | 0 | 1 | 1 | 1 | 7 |

Table 3.2. Assessment outcomes of included cohort studies

| NOS for Cohort study | Selection | | | | Comparability | | Exposure | | | Total score |
| --- | --- | --- | --- | --- | --- | --- | --- | --- | --- | --- |
|  | Ascertainment of exposure | Exposure represent-  ativeness | Control selection | Outcome wasn't present at start | Controls for alcohol/  drinker | Controls for other factors | Ascertainment of outcome | Follow-up long enough | Adequacy of follow-up |  |
| Li 2022 | 1 | 1 | 1 | 1 | 0 | 1 | 1 | 1 | 1 | 8 |
| He 2021 | 1 | 0 | 1 | 1 | 1 | 1 | 1 | 1 | 1 | 8 |
| Ishida 2021 | 1 | 0 | 1 | 1 | 0 | 0 | 1 | 1 | 1 | 6 |
| Takeno 2021 | 1 | 0 | 1 | 1 | 1 | 0 | 1 | 0 | 1 | 6 |
| Hou 2020 | 1 | 0 | 1 | 1 | 1 | 1 | 1 | 0 | 1 | 7 |
| Kim 2020 | 1 | 1 | 1 | 1 | 1 | 0 | 1 | 0 | 1 | 7 |
| Mizuno 2016 | 1 | 0 | 1 | 1 | 0 | 0 | 1 | 0 | 1 | 5 |
| Oniki 2016 | 1 | 1 | 1 | 1 | 1 | 0 | 1 | 1 | 1 | 8 |
| Qu 2015 | 1 | 0 | 1 | 1 | 1 | 1 | 1 | 0 | 1 | 7 |
| Suzuki 2004 | 1 | 0 | 0 | 1 | 0 | 0 | 1 | 0 | 1 | 4 |
| Takagi 2002 | 1 | 0 | 0 | 1 | 0 | 0 | 1 | 1 | 1 | 5 |
| Murata 2000 | 1 | 0 | 0 | 1 | 0 | 0 | 1 | 0 | 1 | 4 |
| Suzuki 1996 | 1 | 0 | 1 | 1 | 0 | 0 | 1 | 0 | 1 | 5 |

Table S3.3. Assessment outcomes of included cross-sectional studies

| AHRQ for  Cross-sectional study | Clarify the source of data | List of inclusion /exclusion criteria | Time period for identifying patients | Population source/  Coutinuous subjects | Subjective factors | Quality of evaluation | Reasons for excluding | Control confounding factors | Handle lost data | Response rates and data completeness | Follow-up | Total score |
| --- | --- | --- | --- | --- | --- | --- | --- | --- | --- | --- | --- | --- |
| Hisamatsu 2023 | 1 | 1 | 1 | 1 | 1 | 1 | 1 | 1 | 1 | 1 | 1 | 11 |
| Hyashida 2023 | 1 | 1 | 1 | 0 | 1 | 1 | 1 | 1 | 1 | 1 | 0 | 9 |
| Okura 2023 | 1 | 1 | 1 | 0 | 1 | 1 | 1 | 0 | 1 | 1 | 0 | 8 |
| Wang 2020 | 1 | 1 | 1 | 0 | 1 | 1 | 1 | 0 | 1 | 1 | 1 | 9 |
| Zhu 2020 | 1 | 1 | 0 | 1 | 1 | 1 | 1 | 1 | 1 | 1 | 0 | 9 |
| Han 2019 | 1 | 1 | 0 | 0 | 1 | 1 | 1 | 0 | 1 | 1 | 0 | 7 |
| Xia 2019 | 1 | 1 | 0 | 0 | 1 | 1 | 1 | 0 | 1 | 1 | 0 | 7 |
| Ma 2017 | 1 | 1 | 1 | 1 | 1 | 1 | 1 | 1 | 1 | 1 | 0 | 10 |
| Yin 2016 | 1 | 1 | 1 | 1 | 1 | 1 | 1 | 1 | 1 | 1 | 0 | 10 |
| Idewaki 2015 | 1 | 1 | 1 | 0 | 1 | 1 | 1 | 0 | 1 | 1 | 0 | 8 |
| Yokoyama 2015 | 1 | 1 | 1 | 0 | 1 | 1 | 1 | 1 | 1 | 1 | 0 | 9 |
| Yokoyama 2013 | 1 | 1 | 1 | 0 | 1 | 1 | 1 | 0 | 1 | 1 | 0 | 8 |
| Dakeshi 2008 | 1 | 1 | 1 | 1 | 1 | 1 | 1 | 0 | 1 | 1 | 0 | 9 |
| Nagasawa 2007 | 1 | 1 | 1 | 1 | 1 | 1 | 1 | 1 | 1 | 1 | 0 | 10 |
| Xu 2007 | 1 | 1 | 0 | 0 | 1 | 1 | 1 | 1 | 1 | 1 | 0 | 8 |
| Saito 2003 | 1 | 1 | 0 | 0 | 1 | 1 | 1 | 1 | 1 | 1 | 0 | 8 |
| Amamoto 2002 | 1 | 1 | 0 | 0 | 1 | 1 | 1 | 1 | 1 | 1 | 0 | 8 |
